# Climate-smart forestry through innovative wood products and commercial afforestation and reforestation on marginal land

**DOI:** 10.1073/pnas.2221840120

**Published:** 2023-05-30

**Authors:** Bingquan Zhang, Kai Lan, Thomas B. Harris, Mark S. Ashton, Yuan Yao

**Affiliations:** ^a^Center for Industrial Ecology, Yale School of the Environment, Yale University, New Haven, CT 06511; ^b^The Forest School, Yale School of the Environment, Yale University, New Haven, CT 06511

**Keywords:** afforestation, reforestation, marginal land, greenhouse gas, wood product

## Abstract

Afforestation and reforestation (AR) on marginal land are nature-based solutions to climate change. There is a gap in understanding the climate mitigation potential of protection and commercial AR with different combinations of forest plantation management and wood utilization pathways. Here, we fill the gap using a dynamic, multiscale life cycle assessment to estimate one-century greenhouse gas (GHG) mitigation delivered by (both traditional and innovative) commercial and protection AR with different planting density and thinning regimes on marginal land in the southeastern United States. We found that innovative commercial AR generally mitigates more GHGs across 100 y (3.73 to 4.15 Giga tonnes of CO_2_ equivalent (Gt CO_2_e)) through cross-laminated timber (CLT) and biochar than protection AR (3.35 to 3.69 Gt CO_2_e) and commercial AR with traditional lumber production (3.17 to 3.51 Gt CO_2_e), especially in moderately cooler and dryer regions in this study with higher forest carbon yield, soil clay content, and CLT substitution. In a shorter timeframe (≤50 y), protection AR is likely to deliver higher GHG mitigation. On average, for the same wood product, low-density plantations without thinning and high-density plantations with thinning mitigate more life cycle GHGs and result in higher carbon stock than that of low-density with thinning plantations. Commercial AR increases the carbon stock of standing plantations, wood products, and biochar, but the increases have uneven spatial distributions. Georgia (0.38 Gt C), Alabama (0.28 Gt C), and North Carolina (0.13 Gt C) have the largest carbon stock increases that can be prioritized for innovative commercial AR projects on marginal land.

## Significance

Afforestation and reforestation (AR) are nature-based solutions to climate change. However, the greenhouse gas (GHG) mitigation efficacy of protection or commercial AR is under debate. This study develops a dynamic life cycle assessment to quantify the GHG mitigation potential of protection and commercial AR on marginal land in the southeastern United States. We found that commercial AR with cross-laminated timber and biochar production generally mitigates more GHGs across 100 y than protection AR and commercial AR with traditional lumber production. Protection AR could mitigates more GHGs in a shorter timeframe (≤50 y). These results highlight the role of synergizing protection AR, innovative wood utilization, and strategic forest plantation management in supporting short- and long-term climate change mitigation goals.

The Intergovernmental Panel on Climate Change highlights afforestation and reforestation (AR) as a critical land-based greenhouse gas (GHG) mitigation strategy to limit global warming to 1.5 to 2 °C above preindustrial levels (1–3). However, social and economic competition with other land use (e.g., agriculture and pasture) often limits AR implementation, making “marginal lands” promising candidates (4). The definition of marginal land differs in studies but generally includes abandoned or degraded agricultural and pasture lands, grasslands, shrublands, nonstocked forests, and postburn landscapes. These lands comprise low fertility and/or steeply sloped erosion-prone soils, making them incompatible with other land use but suitable for forest regrowth (4–10). Forests can be restored on marginal land for either protection/conservation or industrial purposes that supply timber and raw materials for wood products (4, 11, 12) or bioenergy (5–8). The global wood product demand is growing rapidly (13). Emerging wood products (e.g., mass timber) have the potential to mitigate climate change by replacing carbon-intensive construction materials (e.g., concrete and steel) (14–17). The source of wood is important. Sourcing timber from existing natural forests negatively impacts biodiversity and other ecosystem services. Commercial AR on marginal land is a promising option to supply timber without creating land competition and converting natural forests.

The GHG mitigation potential of commercial and protection AR has been highlighted in the literature (3, 18–25), but their GHG mitigation efficacy has been debated (18, 26, 27). For instance, commercial AR for wood production in tropical regions is less favorable than protection AR for carbon removal (26), but commercial AR in temperate regions show greater GHG mitigation potential (18). GHG mitigation efficacy of AR depends on regional contexts, such as local climate conditions (18, 26), forest plantation management strategies (3, 19, 27–30), tree species (28, 31), and wood product types and their life cycle activities [e.g., manufacturing processes, use phase, substitution benefits, and end of life (EOL)] (18, 19, 29, 30). Emerging wood products, such as cross-laminated timber (CLT), have more environmental benefits than traditional lumber due to their longer lifetime and higher material substitution credits (replacing concrete and steel) (14–17). Combining wood products with other nature-based solutions, such as biochar and bioenergy derived from forest plantation residues, shows additional carbon benefits by reducing wildfire hazard and providing renewable energy and carbon storage (19). Recent research on circular economy applications to the forest sector revealed the benefits of utilizing waste woody biomass in enhancing resource efficiency and reducing environmental burdens associated with current practices such as prescribed burning (32).

There is a gap in understanding the role of synergizing different, innovative wood uses from AR and forest plantation management strategies in mitigating climate change. Previous studies have compared protection and commercial forest plantations for traditional wood products (18) or compared different product combinations in existing forests (19). Most life cycle assessment (LCA) studies of innovative wood use, such as CLT (33–35) and biochar (36–40), have focused on process- or product-level understandings, and they used traditional, static methods without considering spatial and temporal factors (e.g., forest growth, climate and soil conditions). Global analyses of mass timber products show the GHG mitigation potential of wood use in buildings (16) and their impacts on land use change (41), but high-resolution, spatially explicit understandings of various combinations of wood use and forest plantation management have not been fully explored. Such understandings are critical for region-specific decision-making because of the high spatial heterogeneity of forest carbon pools, e.g., soil organic carbon (SOC), wood products, and the hyperlocal wood markets where the majority of wood harvested is delivered to mills within 100 miles in the southeastern United States (42).

This study addresses these knowledge gaps by dynamic, multiscale LCA modeling (*SI Appendix*, Fig. S1) that links process-scale LCA for wood products and biochar with regional-scale modeling of forest ecosystems on an annual basis for 100 y. Spatially explicit climate and soil conditions were modeled for loblolly pine AR (*Pinus taeda* L.) on marginal land in the southeastern United States at 1 km resolution, including Virginia (VA), Tennessee (TN), North Carolina (NC), Mississippi (MS), Alabama (AL), Georgia (GA), South Carolina (SC), and Florida (FL). The cradle-to-grave LCA includes GHG emissions of forest operations, manufacturing, EOL, carbon sequestration of forests, and material substitution credits for replacing steel and concrete in the construction industry. Emissions from SOC with spatial heterogeneity were simulated in the RothC model (43). Carbon sequestration through forest growth was estimated based on loblolly pine growth in a 25-y rotation using gridded site index data and the 1996 Plantation Management Research Cooperative whole-stand growth and yield model (44).

A scenario analysis was conducted to compare the life-cycle GHG mitigation potentials between protection and commercial AR and to explore the effects of different synergies between forest plantation management (e.g., plantation density and thinning) and the use of harvested wood (e.g., traditional lumber, CLT, and biochar) and forest plantation residues (including aboveground parts of snags, aboveground components of removed trees from thinning, and logging residues). Two baselines were established for protection AR without forest plantation management at low (450 trees/acre) and high plantation densities (900 trees/acre). Nine scenarios were simulated as a combination of three scenarios for different wood and residue uses and three scenarios of varying forest plantation management strategies. This study estimated the carbon stock change as the change from year zero on marginal land where the aboveground and soil carbon stock is assumed to be zero and current state, respectively. Carbon stock changes were estimated for different locations and years to understand their temporal–spatial distributions.

## Results

### 100-Y GHG Balances.

**Fig. 1.**
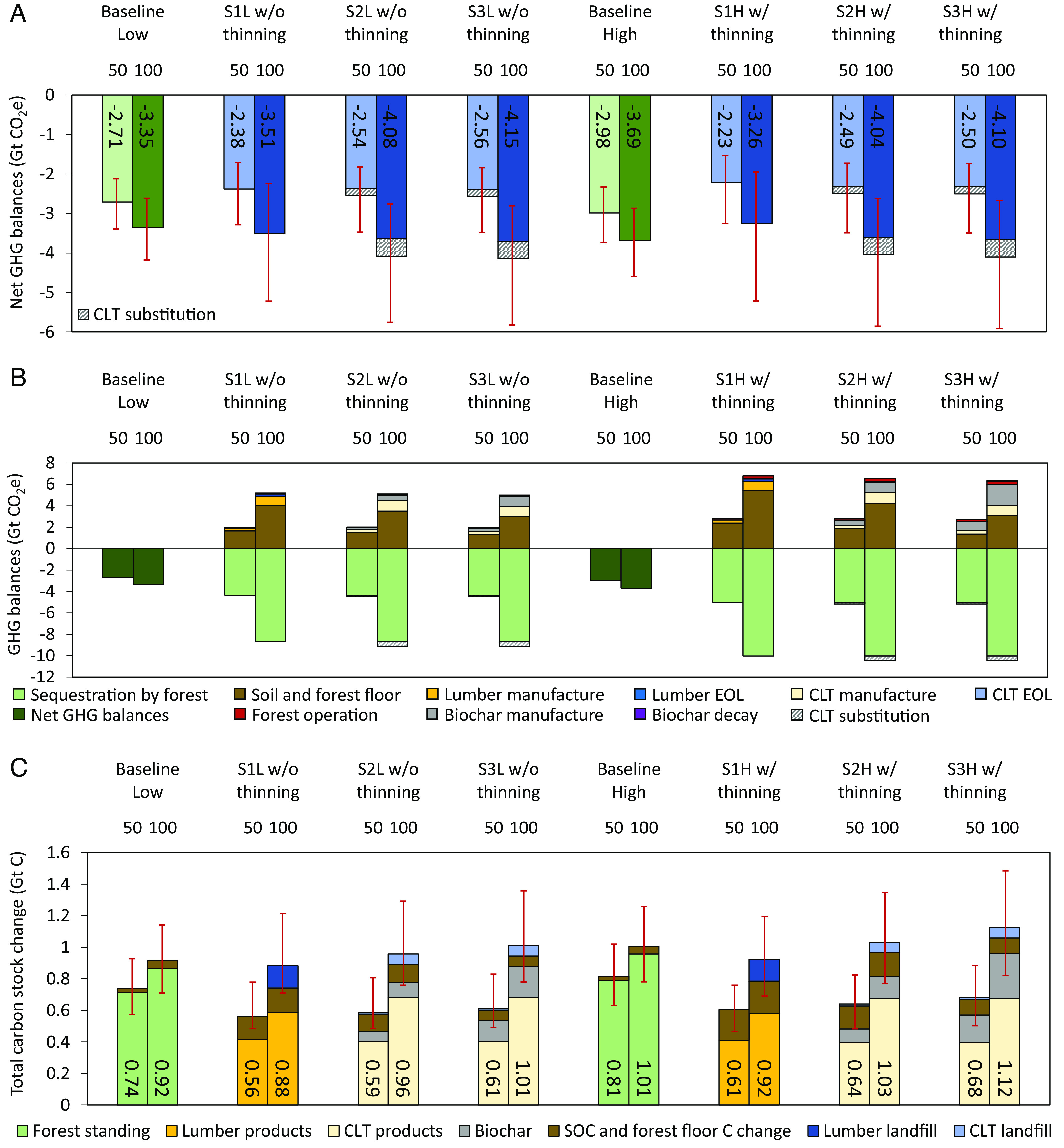
Net GHG balances, GHG balance breakdown by life cycle stages, and total carbon stock change breakdown by carbon pools of AR on marginal land in the southeastern United States at the end of year 100. Net GHG balances under low-density scenarios (S1-3 with or without thinning, L: low density) and high-density scenarios (S1-3 with or without thinning, H: high density) (*A*). GHG balance breakdown by life cycle stages under low-density scenarios and high-density scenarios (*B*). Total carbon stock breakdown by carbon pools under low-density scenarios and high-density scenarios (*C*). Note: the GHG emissions of CLT EOL, biochar decay, and forest operation in figures (*B*) are not visible due to their minimal value compared to others. The maximum ranges of modeled results from uncertainty analysis are shown by the error bars in red. Bars are plotted based on the average values. Numbers in bar charts stand for the average values of the total net GHG balances and total carbon stock change in figure (*A* and *C* ), respectively. The dark green bars for baselines in figure (*B*) represent net GHG balances. Detailed calculations and explanations are documented in *SI Appendix*, *SI Note 1*.

Fig. 1 shows that for low-density plantations without thinning, Scenario 3 (S3: innovative commercial AR for CLT production with 100% residue removal for biochar production, a conceptual, extreme case) has similar ranges of 100-y GHG mitigation potential with Scenario 2 (S2: innovative commercial AR for CLT production with 50% residue removal for biochar production), both of which likely deliver more GHG mitigations than those of Scenario 1 (S1: commercial AR for lumber production without plantation residue removal) and baseline (protection AR). The results of S2 and S3 in low-density plantations without thinning are also similar to the results of the same scenarios in high-density plantations, because more CO_2_ sequestrated by high-density plantations are primarily canceled out by greater emissions from biochar manufacturing and soil and ground surface respiration is associated with more biomass generated in high-density plantations (Fig. 1*B*). On average, high-density baseline protection AR has higher GHG mitigation potential than high-density S1 with thinning, because residues from thinning are left on soil and forest floor, which contributes to GHG emissions through biomass decomposition (Fig. 1*B*). These GHG emissions are greatly reduced in S2 and S3 as residues are removed for biochar production, resulting in higher average GHG mitigation of S2 and S3 than that of high-density protection AR (Fig. 1 *A* and *B*). In shorter timeframes (e.g., 50 y in Fig. 1), protection AR mitigates more GHG than commercial AR, which can be explained by fast forest growth in early years and the dramatically decreased growth after 50 y for protection AR.

The uncertainty analysis indicates the possibility of higher GHG mitigation of commercial AR than protection AR for both low- and high-density plantations (see the lower bounds of ranges in Fig. 1*A*). These lower bounds reflect optimistic conditions (as defined in *SI Appendix*, *SI Note 9*) with lower temperature, precipitation, and landfill decay rate and higher forest carbon yield (Mg C/ha), mill waste recovery rate, soil clay content, and steel usage for traditional buildings (leading to higher material substitution benefits) (see *SI Appendix*, Tables S1 and S2 for uncertainty analysis inputs). Under the pessimistic conditions, protection AR mitigates slightly more GHGs than those of commercial AR in the high-density plantations but not low-density plantations (see the upper bounds of ranges in Fig. 1*A*). Commercial AR has greater ranges of GHG mitigation potential than those of protection AR given the additional uncertainty of wood product life cycles (contribute to 12 to 15% of variations, see *SI Appendix*, Table S3). However, protection AR may have larger uncertainties because it is more prone to exposure for longer periods of time to natural disturbances such as insects, diseases, wind, and fire (45), which are not included in this study.

The largest GHG mitigation of S2 and S3 is mainly attributed to CLT substitution and biochar, e.g., CLT substitution accounts for 11% of the total GHG mitigation potential in S2 and S3 (Fig. 1*A*). Biogenic carbon emissions through biomass decomposition from soil and the plantation ground are the largest GHG emission contributor (Fig. 1*B*). Converting tree plantation residues to biochar reduces residues left on the plantation floor and soil (zero, 50%, 100% left in S3, S2, and S1, respectively) and therefore reducing emissions from biomass decomposition. Biochar decays slowly (*SI Appendix*, *SI Note 8* and Table S4) and decay emissions are minor (Fig. 1*B*). Biochar also contributes to carbon stock increase (Fig. 1*C*). CLT and biochar production brings more manufacture-related GHG emissions (light yellow and gray bars) than lumber production (golden bars in Fig. 1*B*); however, such emission increases are lower than the emission reduction benefits of biochar (brown bars). This result highlights the benefits of removing and converting plantation residues to biochar for GHG mitigation, although the removal rate needs further ecological considerations for maintaining long-term site productivity (see *Discussion* Section).

Another key factor is CLT substitution, one of the uncertainty sources. Without CLT substitution credits, the comparative conclusions depend on plantation density and thinning practices (*SI Appendix*, Fig. S2 *C* and *D*). Three density scenarios are 1) low-density plantation (450 trees/acre) without thinning, 2) low-density plantation (450 trees/acre) with thinning 25% of standing in year 10, and 3) high-density (900 trees/acre) with thinning 50% of standing in year 10. On average, without substitution credits, high-density protection AR across 100 y has similar GHG mitigation potentials with innovative commercial AR in S2 and S3 (*SI Appendix*, Fig. S2*D*). These comparative trends are similar for low-density plantations with thinning; however, innovative commercial AR without thinning in S2 and S3 mitigates more GHG than that of the baseline protection AR on average, even without substitution credits (*SI Appendix*, Fig. S2*C*). Considering uncertainty, without CLT substitution, all commercial AR in S2 and S3 still mitigate more GHG than that of protection AR regardless of densities under optimistic conditions, while this is not the case under pessimistic conditions (*SI Appendix*, Fig. S2 *C* and *D*).

**Fig. 2.**
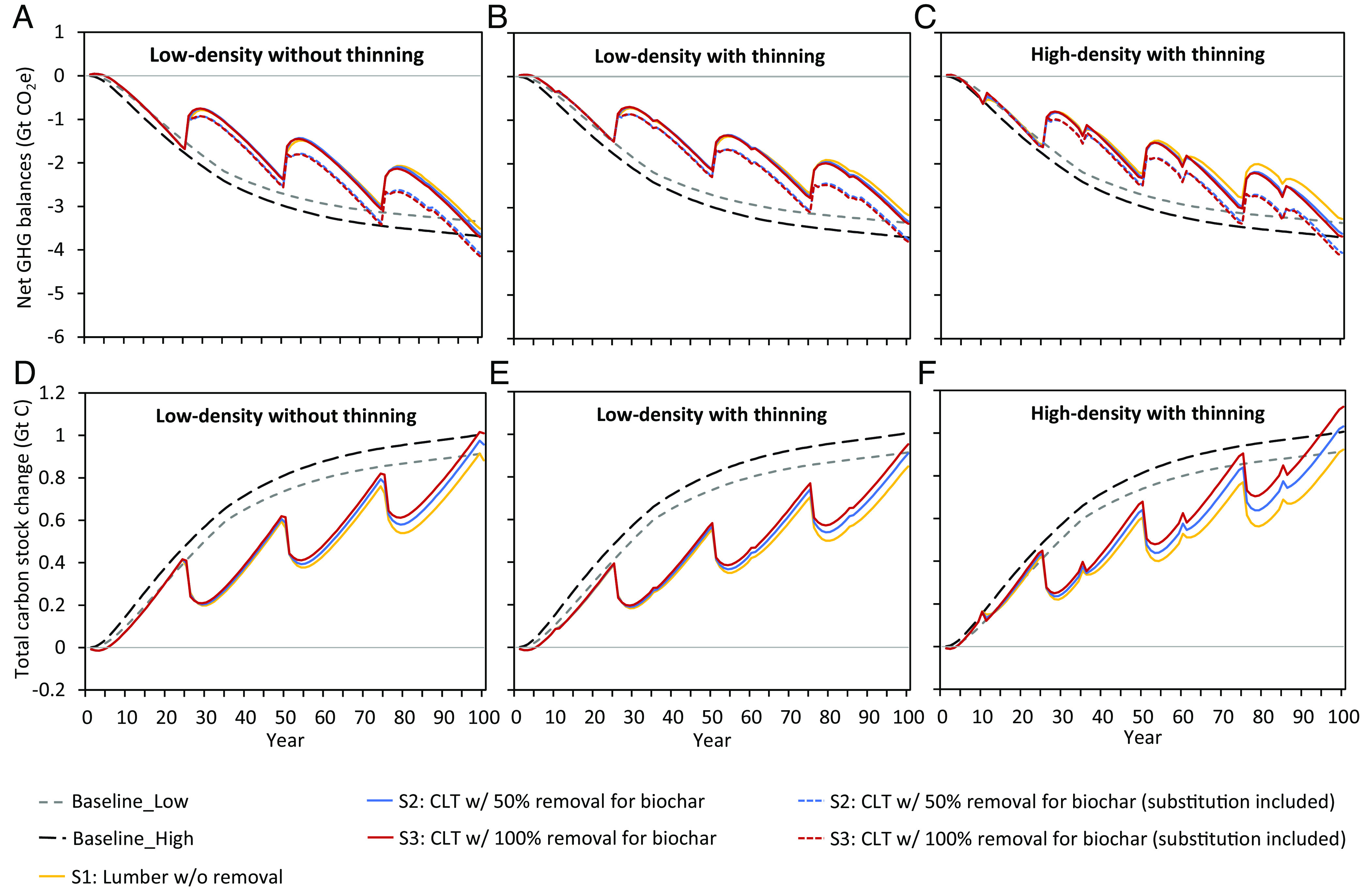
Net GHG balances and total carbon stock change by different scenarios of AR on marginal land in the southeastern United States over 100 y under different scenarios. Net GHG balances: (*A*) low-density without thinning, (*B*) low-density with thinning, (*C*) high-density with thinning. Total carbon stock change: (*D*) low-density without thinning, (*E*) low-density with thinning, (*F*) high-density with thinning. These figures are based on average values.

The climate preferability of commercial AR depends on time, e.g., the GHG mitigation potential of S3 including substitution does not exceed the baseline until year 73 and then crosses the baseline several times (Fig. 2*A*). This can be explained by the temporal changes of GHG emissions and CO_2_ sequestration (*SI Appendix*, Fig. S3). Thinning low-density plantations delays the time when commercial AR reaches similar GHG mitigation potential as the low-density baseline (Fig. 2*A* versus Fig. 2*B*). Besides, thinning a low-density plantation reduces the total 100-y forest carbon sequestrated compared to no thinning (*SI Appendix*, Fig. S2). The total carbon stock change shows similar trends as the net GHG balances (Fig. 2 *D*–*F*).

Overall, the climate change mitigation potential of different AR depends on time, environmental conditions, and material substitution. In the short-term (50 y or shorter) and pessimistic conditions in warmer and more humid climates within our study region with lower forest carbon yield, soil clay content, and CLT substitution, protection AR is likely to be climate favorable. In general and optimistic conditions in climates that are moderately cooler and dryer within the region of our study with higher forest carbon yield, soil clay content, and CLT substitution, innovative commercial AR is likely to achieve greater GHG mitigation than that of protection AR and commercial AR with lumber production in the long term (100 y). Without CLT material substitution benefits, compared to protection AR with the same plantation density, low-density commercial AR without thinning is still climate favorable on average and optimistic conditions, while high-density commercial AR with thinning is preferred only under optimistic conditions.

### 100-Y Carbon Stocks of Different Carbon Pools.

The carbon stock results in Fig. 1*C* highlight the role of AR in increasing the carbon stock of marginal lands. On average, high-density plantations with thinning result in greater carbon stocks than those of low-density plantations but have larger uncertainty ranges in S2 and S3, because of more collected biomass materials from removed trees during thinning in high-density plantations. In total, high-density plantations in the southeastern United States can contribute to on average 1.01 (range: 0.78 to 1.26), 0.92 (0.69 to 1.19), 1.03 (0.77 to 1.35), and 1.12 (0.82 to 1.48) Gt of carbon storage over 100 y under baseline, S1, S2, and S3, respectively (Fig. 1*C*).

Thinning affects carbon stocks through its impact on biomass availability. Thinning high-density plantations generates residues for biochar that accounts for 14.0 to 25.6% of total carbon stock (Fig. 1*C*). Without thinning, biochar contributions in low-density scenarios are much lower (10.3 to 19.4% in Fig. 1*C*). Thinning low-density plantations does not yield higher carbon stocks (Fig. 2 *D* and *E*) because of the lower production of wood products and biochar (*SI Appendix*, Fig. S2*E*). This finding is consistent with other studies indicating that thinning can lower the total standing volume in low-density plantations (46, 47).

**Fig. 3.**
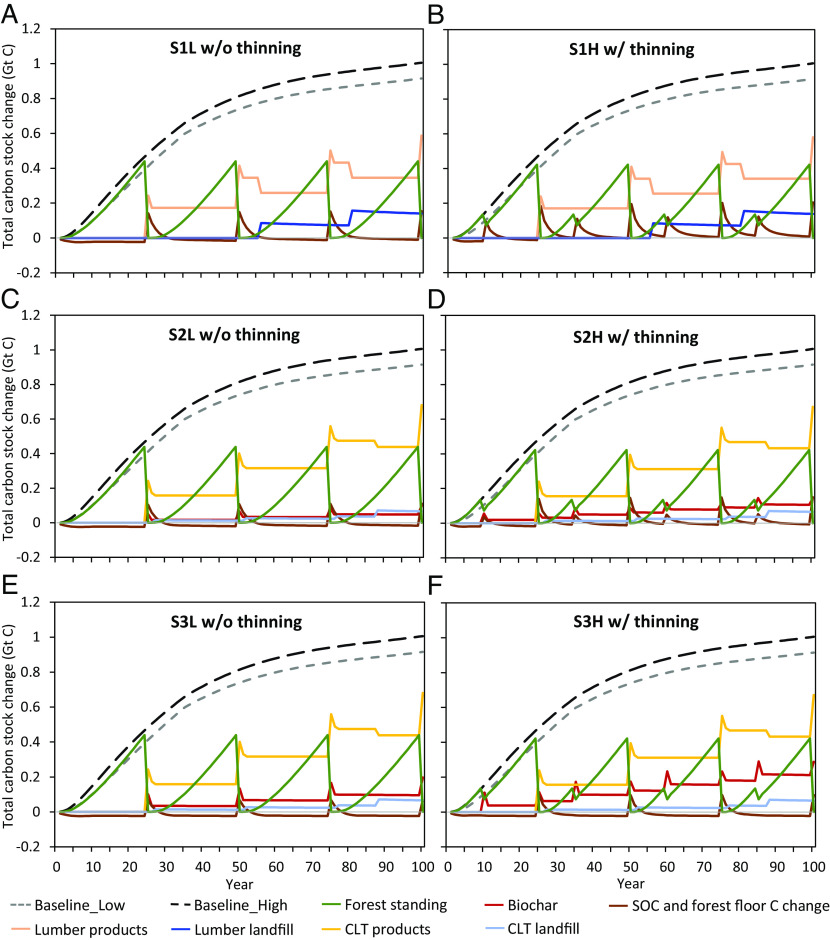
Total carbon stock change breakdown by carbon pool AR on marginal land in the southeastern United States over 100 y under different scenarios. (*A*) Low density without thinning—lumber without plantation residue removal, (*B*) high density with thinning—lumber without plantation residue removal, (*C*) low density without thinning—CLT with 50% plantation residue removal for biochar, (*D*) high density with thinning—CLT with 50% plantation residue removal for biochar, (*E*) low density without thinning—CLT with 100% plantation residue removal for biochar, and (*F*) high density with thinning—CLT with 100% plantation residue removal for biochar. These figures are based on average values.

In general, high-density plantations with thinning and 100% residue removal yield the highest carbon stock. Commercial plantations demonstrate stable growth in carbon stock every 25 y, whereas protection plantations experience a slowing increase in carbon stock after year 50 (Fig. 2 *D*–*F*). Carbon sequestered by trees mainly goes to wood products after the first rotation (year 25) in commercial plantations (Fig. 3, detailed carbon flows are shown in *SI Appendix*, Fig. S4). From years 55 and 85, a portion of wood product carbon moves to landfills when lumber and CLT reach their lifetime (lumber: 30 y; CLT: 60 y). A small portion of carbon in litterfall, root, and plantation residues moves to SOC. The remaining sequestered carbon in plantation residues under S2 and S3 goes to biochar. Compared to protection AR, commercial AR for traditional lumber production has limited capacity to increase carbon storage further, as demonstrated by similar 100-y carbon stock results of S1 and baselines (Fig. 1*C*). CLT scenarios have greater carbon stock on average than lumber scenarios because of the high carbon stock in CLT and biochar due to their longer lifetime and residence time, respectively (*SI Appendix*, Fig. S4). The total amount of carbon stored in CLT and landfill is slightly higher than the carbon stored in lumber and its landfill over 100 y due to the shorter lifetime of lumber (Fig. 1*C*). The modeled SOC content increases less in the CLT cases compared to the lumber cases over 100 y because residue removal in the CLT cases results in less plant carbon input to the soil (Fig. 3). However, biochar contributes to a much higher total carbon content in the soils over 100 y in CLT cases compared to the lumber cases (Fig. 3) because biochar carbon has a much longer retention time than SOC (*SI Appendix*, *SI Note 8*). It indicates that higher residue removal rate for biochar production results in an overall greater carbon stock increase in soil.

Overall, innovative commercial AR with CLT and biochar demonstrate considerably more carbon stock increases than those of protection AR and commercial AR with traditional lumber production over 100 y on average and optimistic conditions (in climates that are moderately cooler and dryer within the region of our study with higher forest carbon yield, soil clay content, and CLT substitution). Protection AR has similar or lower carbon stock increase as innovative commercial AR on pessimistic conditions (in climates that are warmer and more humid within the region of our study with lower forest carbon yield, soil clay content, and CLT substitution) across 100 y, but greater carbon stock increase in general within a shorter timeframe. This conclusion is consistent across all scenarios and timeframes (see *SI Appendix*, Figs. S2 and S5 for more details).

### Mapped Carbon Stock Change over 100 Y.

**Fig. 4.**
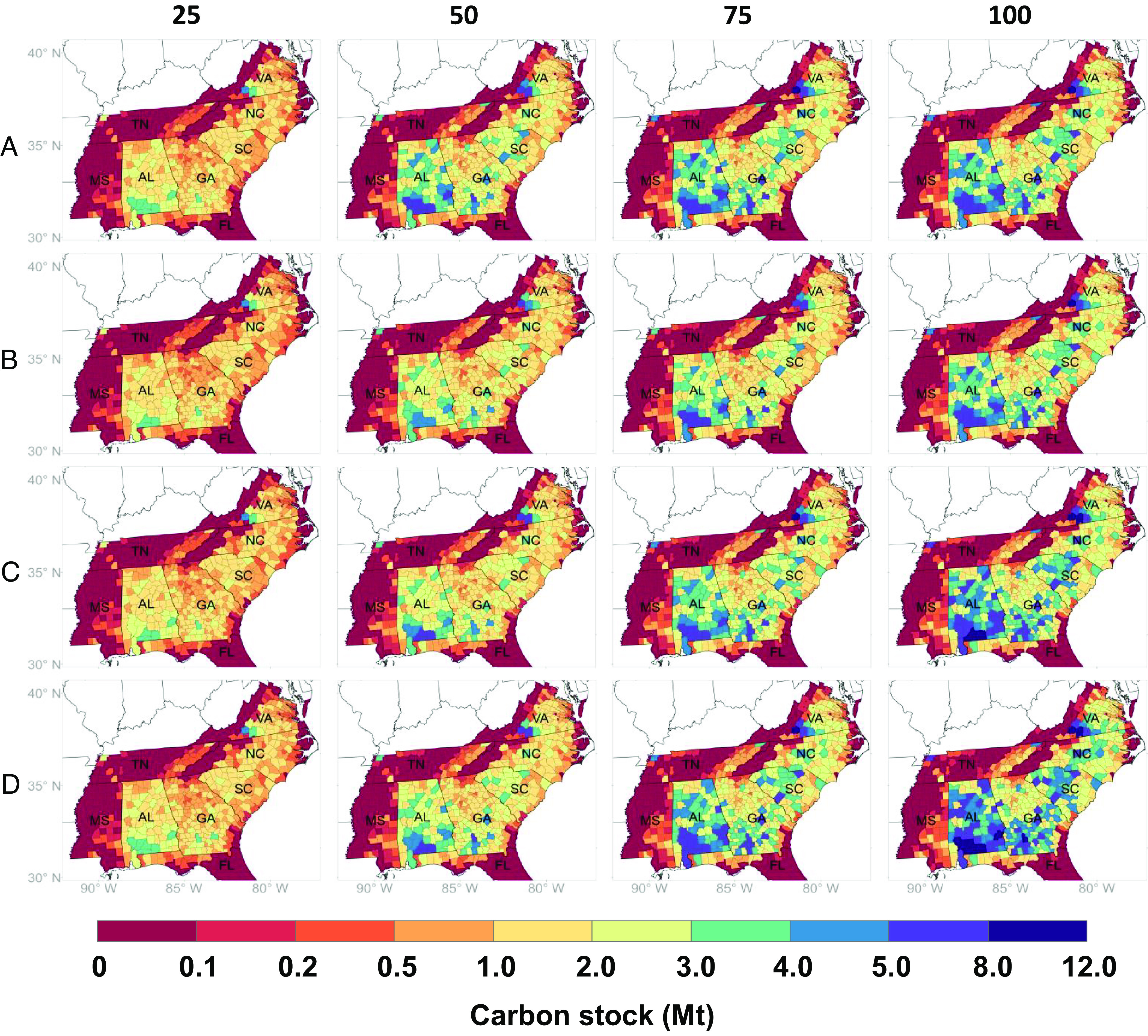
Mapped aggregated carbon stock change from AR on marginal land at county level under high density with thinning scenarios in the southeastern United States in years 25, 50, 75, and 100. (*A*) Baseline, (*B*) S1—lumber without forest residue removal, (*C*) S2—CLT with 50% forest residue removal for biochar, (*D*) S3—CLT with 100% forest residue removal for biochar. VA: Virginia, TN: Tennessee, SC: South Carolina, NC: North Carolina, MS: Mississippi, GA: Georgia, AL: Alabama, FL: Florida. Note: The color changes in some areas along state boundaries between TN and AL, MS and AL, and GA and FL are abrupt due to the low availability of site index data of loblolly pine in TN, MS, and FL (*SI Appendix*, Fig. S6*A*). These figures are based on average values.

The aggregated carbon stock change on marginal land for each county across 100 y in the southeastern United States shows temporal–spatial variations under different scenarios (Fig. 4). Positive values indicate carbon stock increases compared with year 0 where carbon stocks are assumed to be zero on marginal land. In year 25, most counties are red and orange, indicating a slight carbon stock increase of 0 to 2 Mt C per county. Some counties in AL, GA, SC, and VA turn blue by year 50, indicating moderate carbon stock increase by 2 to 5 Mt C per county. By years 75 and 100, more counties in blue and purple indicate substantial carbon stock increases (5 to 12 Mt C).

The spatial pattern of carbon stock increase (Fig. 4) is driven by variations in site index and marginal land availability. For example, some regions (dark red) have minor carbon stock increases across all years, including MS, TN, northern FL, western VA, and NC, because of their low marginal land availability and site index (*SI Appendix*, Fig. S6). The impacts of site index and marginal land availability differ by region. Eastern SC (the lower coastal plain) has a higher site index than its western areas (the piedmont and upper coastal plain) (*SI Appendix*, Fig. S6*A*), but western SC yields more carbon stock increases because of the greater availability of marginal land (*SI Appendix*, Fig. S6*B*). Another example is the piedmont of southwestern VA and eastern TN where marginal lands have similar availability, suggesting similar distributions of their site index and carbon stock increases. At the state level, GA achieves the highest 100-y total carbon stock increase (0.38 Gt C) in high-planting density with thinning scenarios, followed by AL (0.28 Gt C) and NC (0.13 Gt C) (*SI Appendix*, Table S5). These three states account for 70.5% of the total carbon stock increase of the entire study region. At the regional level, southern GA, southern AL, and southwestern VA (dark purple) demonstrate substantial 100-y carbon stock increase because of their high marginal land availability and high site index (*SI Appendix*, Fig. S6). These regions can be priorities for commercial AR on marginal land.

## Discussion

This study highlights the importance of strategically considering the combination of available marginal land, AR management, and sustainable wood products as a valid nature-based solution. Our dynamic LCA results suggest that commercial AR on marginal land offers benefits in supplying timber for wood products, mitigating climate change, and establishing a long-term carbon sink. Based on our analysis, commercial AR on marginal land in the southeastern United States can supply up to 485.2 million oven dry metric tons of loblolly pine logs for a 25-y rotation. This amount is around nine times of the total annual harvested loblolly pine timber provided by the region (*SI Appendix*, Table S6). This additional timber can meet future demands of wood products without converting natural forests to plantations or more intensive management and without losing productive agricultural farmland.

The extent of GHG mitigation benefits depends on wood product, planting density, thinning practices, time, and climate and environmental conditions. Protection AR was estimated to have 10.1 Gt CO_2_e y^−1^ GHG mitigation potential from 678 Mha of land globally in 2030 (equivalent to 14.9 Mt CO_2_e y^−1^ per Mha) (26). Our findings show a similar potential with an average of 0.03 Gt CO_2_e y^−1^ (with 2.1 Mha potential marginal land, equivalent to 14.3 Mt CO_2_e y^−1^ per Mha) for protection AR. Commercial AR for traditional lumber production shows lower or similar average values of GHG mitigation potential and carbon stock increase with larger uncertainty ranges compared to protection AR, although this study does not model natural disturbances and possible carbon losses resulting from greater exposure to natural disturbances with prolonged forest stocking in protection AR. Commercial AR with CLT and biochar using high-density plantations with thinning and low-density without thinning are more likely to achieve higher long-term GHG mitigation potential than that of protection AR, especially in moderately cooler and dryer regions in this study with higher forest carbon yield (higher site index region); soil clay content; CLT substitution benefit; and less GHG emissions from soil, forest floor, and landfill. But, this is not the case in the shorter term (e.g., 50 y) or warmer and more humid regions with lower forest carbon yield and soil clay content in this study. Given the urgency of GHG mitigation, a mix of protection and innovative AR could be useful to meet near-term climate change mitigation goals while providing a propeller for driving longer-term GHG reduction and carbon storage through innovative wood products and biochar. Considering variations of net GHG balances, commercial AR is more climate favorable than protection AR in moderately cooler and less moist regions with higher site index and soil clay content. Additionally, this study pinpoints the regions where innovative AR can deliver higher carbon stock increase than traditional commercial AR and protection AR in general conditions, including most of Alabama and the Piedmont of North Carolina, South Carolina, Georgia, and central southern Virginia, and the Red Hills and Sandhill regions of Georgia and South Carolina. Other economic and social factors related to protection and commercial AR should also be considered for region-specific decision-making.

Our results demonstrate the critical contribution that material substitution can make to maximizing the GHG mitigation potential of innovative wood products. Securing the replacement of carbon-intensive materials such as steel and concrete is essential to reduce GHG and increase carbon stock in buildings (14–17). Although we do not directly model rebound effects and market factors (e.g., price competitiveness between CLT and steel), we evaluated the impacts of variations in material substitution rates. We also do not consider the future decarbonization of steel and concrete. However, even without material substitution, our results show a high possibility of greater GHG mitigation by innovative commercial AR in low-density plantations without thinning than that of protection AR, though likely not for high plantation density. Future research should consider these factors, such as recent studies (48–50), although none of the previous studies have explored AR on marginal land.

Interestingly, higher plantation residue removal (100% removal) for biochar production contributes to negligible greater GHG mitigation potentials (1.5 to 1.9% more), although higher carbon stock (4.4 to 8.7% more) over 100 y is achived by the 100% removal rate compared with the 50% removal rate. One hundred percent removal is an extreme conceptual scenario to explore the maximum possibility, and the actual forest removal ratio should be lower given ecological considerations of maintaining soil carbon stocks and associated productivity (51–53). Previous literature recommends retaining at least 33 to 50% of plantation residues in the United States (51) and considering ecosystem services (e.g., biodiversity) when developing region-specific plantation residue removal practices (54–56).

One limitation of this study is the exclusion of the potential social and economic aspects discussed in prior marginal land literature (5, 8). The marginal land used in this study is defined based only on biophysical properties and specific land cover types (4). Whether such marginal lands will be reforested to plantations depends on additional socioeconomic and cultural values that landowners have, which can be complementary or in conflict (e.g., hunting, endangered species conservation, recreation, esthetics, food security) (57–59). In addition, economic circumstances must be considered, such as strong demand for local timber carbon markets and policy incentives. Future AR research should explore the social and economic implications and potential trade-offs between meeting climate objectives and delivering ecosystem services. However, in all circumstances, reforestation for commercial uses needs to plan for set-aside areas for protection that diversify at landscape scales to improve ecosystem services (59–61). This study only considers one tree species, given the challenges of forest modeling and spatial allocation of multiple species on marginal land. Multispecies plantations could provide more stable carbon capture in some tropical and temperate regions, and offer additional cobenefits for ecosystem services (25, 31, 62), which should be considered in future LCA when the challenges above can be tackled.

Active forest management in commercial plantations can potentially reduce fire risk by thinning, periodic harvesting, and residue removal (63) and consequently safeguards carbon stocks, especially at an increased risk of natural disturbance occurrence owing to global climate change. Although natural disturbance is not considered in our analysis, this does not change the conclusion as the disturbance will further decrease the GHG mitigation potential and carbon stock of the protection AR in the long term. In addition, this study does not consider the impact of climate change on forest growth in the future and unusual weather conditions that might happen in some regions. Previous studies for the southern United States found growth declines of loblolly pine due to increased temperature (64), but higher productivity for loblolly pine harvested at age 25 due to enriched atmosphere CO_2_ concentration (65). Other studies have generally indicated a strong CO_2_ fertilization on biomass growth of young forest plantations (66, 67) but not mature forest stands (68). As a result, innovative commercial AR with younger trees will be even more climate beneficial than protection AR considering the influences of natural disturbances and climate change, particularly higher CO_2_ concentration. Future research is encouraged to quantify the impacts of climate change on the GHG mitigation efficacy of AR.

## Materials and Methods

### LCA Framework.

In this study, a cradle-to-grave multiscale dynamic LCA framework was developed (*SI Appendix*, Fig. S1), which integrated the Geographic Information System (GIS) model, the forest growth model, SOC model, and process-based models of producing wood products (i.e., lumber, CLT, and biochar). A 100-y cutoff time horizon was adopted to be consistent with climate change literature (69). The multiscale LCA provides insights on product- and regional-scale life cycle environmental implications (32). Details in calculating the net GHG emissions and carbon stock change are documented in *SI Appendix*, *SI Note 1*.

### Identification of Marginal Land in the United States.

Marginal lands in this study included agricultural lands with challenging soils, pasture lands with challenging soils, and nonstocked forest patches, which are three (in total of 2.1 million hectares) out of ten opportunity classes identified by the study (4). Other land classes, such as shrub and protected areas, were excluded as they either naturally exist and/or provide essential ecosystem services. Postburned landscapes and frequently flooded areas were excluded as they are vulnerable to natural disturbance. Commercial AR on urban open spaces may bring disturbances to the urban environment, so urban areas were excluded. The GIS data in 30 m resolution were collected from (70). The selected three marginal land layers for the southeastern United States were downscaled from 30 m to 1 km pixels by the aggregate by sum technique. All spatial data (*SI Appendix*, Table S7) were processed and visualized by ArcMap 10.8.1 (71).

### Forest Growth and Yield.

A growth and yield model (44) was used to simulate forest growth for site-prepared loblolly pine (*Pinus taeda* L.) plantations in the three prominent physiographic regions in the southern United States: piedmont, upper coastal plain, and lower coastal plain. The reliability of the model has been demonstrated (72). A demo simulator was used in this study (73). The inputs include site index, physiographic region, planting density, and other silvicultural treatments (73). The annual total dry mass of a whole tree includes litterfall (needles and wood debris biomass that fall on the soil), foliage (living needle biomass), branches (living branch biomass), stem (above-stump stem outside-bark biomass), and roots (coarse root biomass). The calculations for a specific site index are in *SI Appendix*, *SI Note 2*. All biomass dry mass was transformed to carbon mass using an average carbon content of 50% for wood biomass and foliage (74).

### Forest Operations.

At the beginning of the 25-y rotation, site preparation, herbicide application, and planting are performed (75). During the rotation, fertilizers are applied twice in years 10 and 16. At the end of the rotation, the clear-cut logging harvests standing trees and yields logs (inside-bark), snags, and residues, including foliage and branch. In Scenarios 1 to 3, logs are transported to produce wood products. In Scenarios 2 and 3, collected residues and snags are chipped on forest sites and then transported for biochar production. The application rate and life-cycle GHG emissions of chemicals and fuels used in forest operations are shown in *SI Appendix*, Tables S8 and S9, respectively. The life-cycle GHG emissions of chemicals and fuels were estimated using the ecoinvent 3.6 cutoff database (76), GREET 2021 (77), and literature (77, 78) as documented in *SI Appendix*, Tables S9 and S10.

### Forest SOC Modeling.

The SOC changes were simulated by the RothC model (version 26.3) (70), which has been validated and widely used for forestland (79–89). The model was run on a monthly basis from years to centuries with inputs including monthly carbon inputs, monthly mean temperature, monthly open pan evaporation, monthly precipitation, soil clay content, total SOC content, soil depth, and whether soil is monthly covered (70). The uncollected harvesting residues (including foliage and branches), snags, trees from thinning, roots, and annually generated litterfall are the carbon input to the SOC. The data of soil clay content and total SOC content are from ISRIC-World Soil Information (90). The monthly climate date is an average calculation from 1991 to 2020 derived from the CRU TS 4.05 dataset (91). The output of RothC includes annual total SOC content (Mg C/ha) and annual CO_2_ emissions (Mg CO_2_/ha). We adopted a method (92) to investigate the impacts of input data uncertainties on RothC results by inputting minimum and maximum values (based on a 95% CI) of predefined input parameters (initial SOC, carbon inputs, temperature, precipitation, and soil clay content) that have the greatest impacts on RothC outputs. More details are available in *SI Appendix*, *SI Note 9*.

### LCA of Traditional Lumber, CLT, and Biochar.

Harvested logs are transported to saw mills that include processes such as debarking, sawing, kiln drying, planing, and power generation (*SI Appendix*, *SI Note 4*) (93, 94). Then, lumber are distributed to markets and finally landfilled after their useful lifespan (95). *SI Appendix*, Table S11 documents the parameters of saw mill models. The upstream GHG emissions were derived from ecoinvent 3.6 database (76) and GREET 2020 (77). The landfill GHG emissions were estimated using (96, 97) with a consideration of energy recovery (*SI Appendix*, *SI Note 5*). CLT has a similar life cycle with lumber but differs in production and EOL. The life cycle inventory data of CLT production are documented in *SI Appendix*, *SI Note 6* and Table S12. Fifty percent of CLT panel is assumed to be recycled (93), while the rest is landfilled. CLT substitution was estimated based on the traditional structural materials used in the same floor area (17) (*SI Appendix*, *SI Note 6*). Logging residues and snags are chipped and sent to a pyrolysis plant for biochar production that was simulated in Aspen Plus (see *SI Appendix*, Fig. S7 for the system diagram and *SI Appendix*, *SI Note 7* for details). The end-of-life GHG emissions of biochar applied as soil amendment are documented in *SI Appendix*, *SI Note 8*. The life cycle carbon balances are shown in *SI Appendix*, Fig. S4. This study deploys a two-step uncertainty analysis for wood product systems (see *SI Appendix*, *SI Note 9* for details).

## Supplementary Material

Appendix 01 (PDF)

## Data Availability

All study data are included in the article and/or supporting information.

## References

[1] J. Roy ., “Sustainable development, poverty eradication and reducing inequalities” in Global Warming of 1.5°C. An IPCC Special Report on the Impacts of Global Warming of 1.5°C above Pre-Industrial Levels and Related Global Greenhouse Gas Emission Pathways, in the Context of Strengthening the Global Response to the Threat of Climate Change, Sustainable Development, and Efforts to Eradicate Poverty, Masson-Delmotte , Eds. (Cambridge University Press, Cambridge, UK and New York, NY, USA, 2018), pp. 445–538, 10.1017/9781009157940.007.

[2] A. Arneth , “Framing and context” in Climate Change and Land: An IPCC Special Report on Climate Change, Desertification, Land Degradation, Sustainable Land Management, Food Security, and Greenhouse Gas Fluxes in Terrestrial Ecosystems, P. R. Shukla , Eds. (Cambridge University Press, Cambridge, UK and New York, NY, USA, 2022), pp. 77–130 10.1017/9781009157988.003.

[3] B. E. Law , Land use strategies to mitigate climate change in carbon dense temperate forests. Proc. Natl. Acad. Sci. U.S.A. **115**, 3663–3668 (2018). 29555758 10.1073/pnas.1720064115 PMC5889652

[4] S. C. Cook-Patton , Lower cost and more feasible options to restore forest cover in the contiguous United States for climate mitigation. One Earth **3**, 739–752 (2020).

[5] M. Khanna , Redefining marginal land for bioenergy crop production. GCB Bioenergy **13**, 1590–1609 (2021).

[6] I. Emery, S. Mueller, Z. Qin, J. B. Dunn, Evaluating the potential of marginal land for cellulosic feedstock production and carbon sequestration in the United States. Environ. Sci. Technol. **51**, 733–741 (2017). 27976872 10.1021/acs.est.6b04189

[7] I. Gelfand , Sustainable bioenergy production from marginal lands in the US Midwest. Nature **493**, 514–517 (2013). 23334409 10.1038/nature11811

[8] C. Jiang, K. Guan, M. Khanna, L. Chen, J. Peng, Assessing marginal land availability based on land use change information in the contiguous United States. Environ. Sci. Technol. **55**, 10794–10804 (2021). 34297551 10.1021/acs.est.1c02236

[9] B. Zhang, A. Hastings, J. C. Clifton-Brown, D. Jiang, A. P. C. Faaij, Modeled spatial assessment of biomass productivity and technical potential of Miscanthus × giganteus, Panicum virgatum L., and Jatropha on marginal land in China. GCB Bioenergy **12**, 328–345 (2020). 10.1111/gcbb.12664 PMC7217200 32421018

[10] K. D. Holl , Redefining “abandoned” agricultural land in the context of reforestation. Front. For. Glob. Chang. **5**, 1–6 (2022). 10.3389/ffgc.2022.933887 PMC12520204 41098808

[11] Y. Yang , Restoring abandoned farmland to mitigate climate change on a full earth. One Earth **3**, 176–186 (2020).

[12] L. E. Nave , Reforestation can sequester two petagrams of carbon in US topsoils in a century. Proc. Natl. Acad. Sci. U.S.A. **115**, 2776–2781 (2018). 29483245 10.1073/pnas.1719685115 PMC5856546

[13] UNECE/FAO, “Chapter 1 Economic overview and policies” in Forest products–Annual market review 2020-2021 (United Nations (UNECE), Geneva, Switherland and FAO, Rome, Italy, 2021).

[14] S. Pei , Cross-laminated timber for seismic regions: Progress and challenges for research and implementation. J. Struct. Eng. **142**, 1–11 (2016).

[15] O. Espinoza, U. Buehlmann, Cross-laminated timber in the USA: Opportunity for hardwoods? Curr. For. Rep. **4**, 1–12 (2018).

[16] G. Churkina , Buildings as a global carbon sink. Nat. Sustain. **3**, 269–276 (2020).

[17] B. D’Amico, F. Pomponi, J. Hart, Global potential for material substitution in building construction: The case of cross laminated timber. J. Clean. Prod. **279**, 123487 (2021).

[18] E. J. Forster, J. R. Healey, C. Dymond, D. Styles, Commercial afforestation can deliver effective climate change mitigation under multiple decarbonisation pathways. Nat. Commun. **12**, 1–12 (2021). 34158494 10.1038/s41467-021-24084-x PMC8219817

[19] B. Cabiyo , Innovative wood use can enable carbon-beneficial forest management in California. Proc. Natl. Acad. Sci. U.S.A. **118**, e2019073118 (2021). 34810238 10.1073/pnas.2019073118 PMC8670525

[20] G. M. Domke, S. N. Oswalt, B. F. Walters, R. S. Morin, Tree planting has the potential to increase carbon sequestration capacity of forests in the United States. Proc. Natl. Acad. Sci. U.S.A. **117**, 24649–24651 (2020). 32958649 10.1073/pnas.2010840117 PMC7547226

[21] K. G. Austin , The economic costs of planting, preserving, and managing the world’s forests to mitigate climate change. Nat. Commun. **11**, 1–9 (2020). 33262324 10.1038/s41467-020-19578-z PMC7708837

[22] J.-F. Bastin , The global tree restoration potential. Science **365**, 76–79 (2019). 31273120 10.1126/science.aax0848

[23] J. E. Fargione , Natural climate solutions for the United States. Sci. Adv. **4**, 1–15 (2018). 10.1126/sciadv.aat1869 PMC6235523 30443593

[24] D. R. Cameron, D. C. Marvin, J. M. Remucal, M. C. Passero, Ecosystem management and land conservation can substantially contribute to California’s climate mitigation goals. Proc. Natl. Acad. Sci. U.S.A. **114**, 12833–12838 (2017). 29133408 10.1073/pnas.1707811114 PMC5715745

[25] B. W. Griscom , Natural climate solutions. Proc. Natl. Acad. Sci. U.S.A. **114**, 11645–11650 (2017). 29078344 10.1073/pnas.1710465114 PMC5676916

[26] S. L. Lewis, C. E. Wheeler, E. T. A. Mitchard, A. Koch, Restoring natural forests is the best way to remove atmospheric carbon. Nature **568**, 25–28 (2019). 30940972 10.1038/d41586-019-01026-8

[27] H. Keith , Managing temperate forests for carbon storage: Impacts of logging versus forest protection on carbon stocks. Ecosphere **5**, art75 (2014).

[28] K. Naudts , Europe’s forest management did not mitigate climate warming. Science **351**, 597–600 (2016). 26912701 10.1126/science.aad7270

[29] C. D. Oliver, N. T. Nassar, B. R. Lippke, J. B. McCarter, Carbon, fossil fuel, and biodiversity mitigation with wood and forests. J. Sustain. For. **33**, 248–275 (2014).

[30] T. W. Hudiburg, B. E. Law, W. R. Moomaw, M. E. Harmon, J. E. Stenzel, Meeting GHG reduction targets requires accounting for all forest sector emissions. Environ. Res. Lett. **14**, 095005 (2019).

[31] A. M. Osuri , Greater stability of carbon capture in species-rich natural forests compared to species-poor plantations. Environ. Res. Lett. **15**, 034011 (2020).

[32] K. Lan, B. Zhang, Y. Yao, Circular utilization of urban tree waste contributes to the mitigation of climate change and eutrophication. One Earth **5**, 944–957 (2022).

[33] Athena Sustainable Materials Institute, “A life cycle assessment of cross-laminated timber produced in Canada” (Athena Sustainable Materials Institute, Ottawa, Canada, 2013).

[34] C. X. Chen, F. Pierobon, I. Ganguly, Life cycle assessment (LCA) of Cross-laminated timber (CLT) produced in Western Washington: The role of logistics and wood species mix. Sustainability **11**, 1278 (2019).

[35] M. Puettmann, A. Sinha, I. Ganguly, Life cycle energy and environmental impacts of cross laminated timber made with coastal douglas-fir. J. Green Build. **14**, 17–33 (2019).

[36] J. Lehmann , Biochar in climate change mitigation. Nat. Geosci. **14**, 883–892 (2021).

[37] M. Owsianiak , Environmental and economic impacts of biochar production and agricultural use in six developing and middle-income countries. Sci. Total Environ. **755**, 142455 (2021). 33049526 10.1016/j.scitotenv.2020.142455

[38] L. Miller-Robbie , Life cycle energy and greenhouse gas assessment of the co-production of biosolids and biochar for land application. J. Clean. Prod. **91**, 118–127 (2015).

[39] A. Tisserant , Life-cycle assessment to unravel co-benefits and trade-offs of large-scale biochar deployment in Norwegian agriculture. Resour. Conserv. Recycl. **179**, 106030 (2022).

[40] E. Struhs, A. Mirkouei, Y. You, A. Mohajeri, Techno-economic and environmental assessments for nutrient-rich biochar production from cattle manure: A case study in Idaho, USA. Appl. Energy **279**, 115782 (2020).

[41] A. Mishra , Land use change and carbon emissions of a transformation to timber cities. Nat. Commun. **13**, 4889 (2022). 36042197 10.1038/s41467-022-32244-w PMC9427734

[42] J. L. Conrad, Costs and challenges of log truck transportation in Georgia, USA. Forests **9**, 650 (2018).

[43] K. Coleman, D. S. Jenkinson, “RothC-26.3–A model for the turnover of carbon in soil” (Rothamsted Research, Harpenden, UK, 2014).

[44] W. M. Harrison, B. E. Borders, 1996 Yield prediction and growth projection for site-prepared loblolly pine plantations in the Carolinas, Georgia, Alabama and Florida PMRC Technical Report, (University of Georgia, Athens, Georgia, USA, 1996).

[45] R. Seidl, M. J. Schelhaas, W. Rammer, P. J. Verkerk, Increasing forest disturbances in Europe and their impact on carbon storage. Nat. Clim. Chang. **4**, 806–810 (2014). 25737744 10.1038/nclimate2318 PMC4340567

[46] J. L. Clutter, E. P. J. Jones, Prediction of Growth After Thinning in Old-Field Slash Pine Plantations (USDA Forest Service Research Paper SE, 1982), **vol. 19**.

[47] R. L. Amateis, Modeling response to thinning in loblolly pine plantations. South. J. Appl. For. **24**, 17–22 (2000).

[48] B. Christian, P. Stefan, Required displacement factors for evaluating and comparing climate impacts of intensive and extensive forestry in Germany. Carbon Balance Manag. **17**, 14 (2022). 36183047 10.1186/s13021-022-00216-8 PMC9526925

[49] T. Myllyviita, S. Soimakallio, J. Judl, J. Seppälä, Wood substitution potential in greenhouse gas emission reduction–Review on current state and application of displacement factors. For. Ecosyst. **8**, 42 (2021).

[50] J. Seppälä , Effect of increased wood harvesting and utilization on required greenhouse gas displacement factors of wood-based products and fuels. J. Environ. Manage. **247**, 580–587 (2019). 31260924 10.1016/j.jenvman.2019.06.031

[51] B. D. Titus , Sustainable forest biomass: A review of current residue harvesting guidelines. Energy. Sustain. Soc. **11**, 1–32 (2021).

[52] J. James , Effects of forest harvesting and biomass removal on soil carbon and nitrogen: Two complementary meta-analyses. For. Ecol. Manage. **485**, 118935 (2021).

[53] D. L. Achat, M. Fortin, G. Landmann, B. Ringeval, L. Augusto, Forest soil carbon is threatened by intensive biomass harvesting. Sci. Rep. **5**, 1–10 (2015). 10.1038/srep15991 PMC4632129 26530409

[54] A. McEwan, E. Marchi, R. Spinelli, M. Brink, Past, present and future of industrial plantation forestry and implication on future timber harvesting technology. J. For. Res. **31**, 339–351 (2020).

[55] D. P. Christian, W. Hoffman, J. M. Hanowski, G. J. Niemi, J. Beyea, Bird and mammal diversity on woody biomass plantations in North America. Biomass Bioenergy **14**, 395–402 (1998).

[56] D. Andrew Scott, T. J. Dean, Energy trade-offs between intensive biomass utilization, site productivity loss, and ameliorative treatments in loblolly pine plantations. Biomass Bioenergy **30**, 1001–1010 (2006).

[57] M. Hurlbert , “Chapter 7 Risk management and decision-making in relation to sustainable development” in *Climate Change and Land: An IPCC Special Report on Climate Change, Desertification, Land Degradation, Sustainable Land Management, Food Security, and Greenhouse Gas Fluxes in Terrestrial Ecosystems*, P. R. Shukla *et al*., Eds. (Cambridge University Press, Cambridge, UK and New York, NY, USA, 2022), 10.1017/9781009157988.009.

[58] S. Luyssaert , Trade-offs in using European forests to meet climate objectives. Nature **562**, 259–262 (2018). 30305744 10.1038/s41586-018-0577-1 PMC6277009

[59] L. Gamfeldt , Higher levels of multiple ecosystem services are found in forests with more tree species. Nat. Commun. **4**, 1340 (2013). 23299890 10.1038/ncomms2328 PMC3562447

[60] S. Fares, Five steps for managing Europe’s forests-Support resilience and promote carbon storage. Nature **519**, 151–153 (2015). 25810187 10.1038/519407a

[61] A. R. Hof, C. C. Dymond, D. J. Mladenoff, Climate change mitigation through adaptation: The effectiveness of forest diversification by novel tree planting regimes. Ecosphere **8**, e01981 (2017).

[62] C. Messier , For the sake of resilience and multifunctionality, let’s diversify planted forests!. Conserv. Lett. **15**, 1–8 (2022).

[63] J. R. González-Olabarria, T. Pukkala, Integrating fire risk considerations in landscape-level forest planning. For. Ecol. Manage. **261**, 278–287 (2011).

[64] J. Huang, B. Abt, K. Georg, S. Ghosh, Empirical analysis of climate change impact on loblolly pine plantations in the southern United States. Nat. Resour. Model. **24**, 445–476 (2011).

[65] H. E. Burkhart , Regional simulations of loblolly pine productivity with CO_2_ enrichment and changing climate scenarios. For. Sci. **64**, 349–357 (2018).

[66] A. P. Walker , Decadal biomass increment in early secondary succession woody ecosystems is increased by CO 2 enrichment. Nat. Commun. **10**, 454 (2019). 30765702 10.1038/s41467-019-08348-1 PMC6376023

[67] R. J. Norby, Ecological and evolutionary lessons from free air carbon enhancement (FACE) experiments. Annu. Rev. Ecol. Evol. Syst. **42**, 181–203 (2011).

[68] M. Jiang , The fate of carbon in a mature forest under carbon dioxide enrichment. Nature **580**, 227–231 (2020). 32269351 10.1038/s41586-020-2128-9

[69] D. Chen , “Chapter 1 Framing,Context, and Methods” In Climate Change 2021: The Physical Science Basis. Contribution of Working GroupI to the Sixth Assessment Report of the Intergovernmental Panel on Climate Change, V.P. Masson-Delmotte ., Eds. Cambridge University Press, Cambridge, United Kingdom and New York, NY, USA, pp. 147–286, 10.1017/9781009157896.003.

[70] The Nature Conservancy, Reforestation Hub. https://www.reforestationhub.org/ (Accessed 18 August 2022).

[71] ESRI (2020), ArcMap (Version10.8.1). [GIS software]. https://www.esri.com/en-us/arcgis/products/arcgis-enterprise/overview.

[72] W. S. Peay, B. P. Bullock, C. R. Montes, Growth and yield model comparisons for mid-rotation loblolly pine (Pinus taeda L.) plantations in the southeastern US. For. An Int. J. For. Res. **95**, 616–633 (2022).

[73] Plantation Management Research Cooperative, PMRC Growth & Yield Simulator. https://pmrc.uga.edu/simulator (Accessed 24 August 2022).

[74] J. E. Smith, L. S. Heath, K. E. Skog, R. A. Birdsey, “Methods for calculating forest ecosystem and harvested carbon with standard estimates for forest types of the United States” (Gen. Tech. Rep. NE-343, U.S. Department of Agriculture, Forest Service, Northeastern Research Station, 2006).

[75] D. Markewitz, Fossil fuel carbon emissions from silviculture: Impacts on net carbon sequestration in forests. For. Ecol. Manage. **236**, 153–161 (2006).

[76] G. Wernet , The ecoinvent database version 3 (part I): Overview and methodology. Int. J. Life Cycle Assess. **21**, 1218–1230 (2016).

[77] Argonne National Laboratory, “The greenhouse gases, regulated emissions, and energy use in technologies (GREET) model” (US Department of Energy, Argonne National Laboratory, Lemont, USA, 2021).

[78] US Environmental Protection Agency, “AP 42, Fifth edition compilation of air pollutant emissions factors” (US EPA, Washington D.C., USA, 2009).

[79] K. Coleman , Simulating trends in soil organic carbon in long-term experiments using RothC-26.3. Geoderma **81**, 29–44 (1997).

[80] P. Falloon, P. Smith, Simulating SOC changes in long-term experiments with RothC and CENTURY: Model evaluation for a regional scale application. Soil Use Manag. **18**, 101–111 (2006).

[81] A. J. Holder , Measured and modelled effect of land-use change from temperate grassland to Miscanthus on soil carbon stocks after 12 years. GCB Bioenergy **11**, 1173–1186 (2019). 31598141 10.1111/gcbb.12624 PMC6774323

[82] M. Dondini, A. Hastings, G. Saiz, M. B. Jones, P. Smith, The potential of Miscanthus to sequester carbon in soils: Comparing field measurements in Carlow, Ireland to model predictions. GCB Bioenergy **1**, 413–425 (2009).

[83] G. Wang, W. Zhang, W. Sun, T. Li, P. Han, Modeling soil organic carbon dynamics and their driving factors in the main global cereal cropping systems. Atmos. Chem. Phys. **17**, 11849–11859 (2017).

[84] J. Meersmans , Estimation of soil carbon input in France: An inverse modelling approach. Pedosphere **23**, 422–436 (2013).

[85] T. G. Morais, R. F. M. Teixeira, T. Domingos, Detailed global modelling of soil organic carbon in cropland, grassland and forest soils. PLoS One **14**, 1–27 (2019). 10.1371/journal.pone.0222604 PMC6752864 31536571

[86] J. Hillier , Greenhouse gas emissions from four bioenergy crops in England and Wales: Integrating spatial estimates of yield and soil carbon balance in life cycle analyses. GCB Bioenergy **1**, 267–281 (2009).

[87] G. Wang, Z. Luo, P. Han, H. Chen, J. Xu, Critical carbon input to maintain current soil organic carbon stocks in global wheat systems. Sci. Rep. **6**, 1–8 (2016). 26759192 10.1038/srep19327 PMC4725856

[88] K. Ranatunga, R. J. Keenan, S. D. Wullschleger, W. M. Post, M. L. Tharp, Effects of harvest management practices on forest biomass and soil carbon in eucalypt forests in New South Wales, Australia: Simulations with the forest succession model LINKAGES. For. Ecol. Manage. **255**, 2407–2415 (2008).

[89] B. Zhang, J. Xu, Z. Lin, T. Lin, P. C. Faaij, Spatially explicit analyses of sustainable agricultural residue potential for bioenergy in China under various soil and land management scenarios. Renew. Sustain. Energy Rev. **137**, 110614 (2021).

[90] ISRIC, “SoilGrids—Global gridded soil information” (ISRIC-World Soil Information, The Netherlands, 2021), (20 October 2021)

[91] I. Harris, T. J. Osborn, P. Jones, D. Lister, Version 4 of the CRU TS monthly high-resolution gridded multivariate climate dataset. Sci. Data **7**, 109 (2020). 32246091 10.1038/s41597-020-0453-3 PMC7125108

[92] G. Peralta , “12 Uncertainty and validation” in *Global soil organic carbon sequestration potential map–GSOCseq v.1.1 technical manual* (FAO, Rome, Italy, 2022), pp. 202–207, 10.4060/cb2462en.

[93] M. E. Puettmann, F. G. Wagner, L. Johnson, Life cycle inventory of softwood lumber from the inland northwest us. Wood Fiber Sci. **42**, 52–66 (2010).

[94] M. R. Milota, C. D. West, I. D. Hartley, Gate-to-gate life-cycle inventory of softwood lumber production. Wood Fiber Sci. **37**, 47–57 (2005).

[95] R. D. Bergman , “Life-cycle energy and GHG emissions for new and recovered softwood framing lumber and hardwood flooring considering end-of-life scenarios” (Res. Pap. FPL-RP-672, U.S. Department of Agriculture, Forest Service, Forest Products Laboratory, Madison, WI, 2013).

[96] R. Pipatti , “Chapter 3: Solid waste disposal” in 2006 IPCC Guidelines for National Greenhouse Gas Inventories, (Institute for Global Environmental Strategies (IGES), Hayama, Japan, 2006), pp. 6.1–6.49.

[97] K. Lan, S. S. Kelley, P. Nepal, Y. Yao, Dynamic life cycle carbon and energy analysis for cross-laminated timber in the Southeastern United States. Environ. Res. Lett. **15**, 124036 (2020).

