# Supplementary material for: Climate-smart forestry through innovative wood products and commercial afforestation and reforestation on marginal land
Source: Proc Natl Acad Sci U S A. 2023 May 30;120(23):e2221840120. doi: 10.1073/pnas.2221840120 (PMC10265990; doi:10.1073/pnas.2221840120)
Supplement: Supplementary file 1 — Appendix 01 (PDF) [file pnas.2221840120.sapp.pdf]

PNAS

Supporting Information for

Climate-smart forestry through innovative wood products and commercial afforestation and reforestation on marginal land

Bingquan Zhang[a, 1], Kai Lan[a,1], Thomas B. Harris[b], Mark S. Ashton[b], Yuan Yao[a,b]

[a]Center for Industrial Ecology, Yale School of the Environment, Yale University, New Haven, CT 06511, USA
[b]The Forest School, Yale School of the Environment, Yale University, New Haven, CT 06511, USA
[1]B. Zhang and K. Lan contributed equally to this work.

Corresponding author: Yuan Yao

Email: y.yao@yale.edu

This PDF file includes:

Supporting Information (SI) Methods including Notes 1 to 9

SI Figures S1 to S10

SI Tables S1 to S17

SI References

Table of Contents

SI Note 1: Summary of Methodology 4

SI Note 2. Forest growth and yield 6

SI Note 3. Forest SOC modeling 8

SI Note 4. Lumber Production 9

SI Note 5. Landfill of wood waste 10

SI Note 6. CLT production and potential substitution benefits 11

SI Note 7. Biochar Production 12

SI Note 8. End-of-life of biochar 13

SI Note 9. Uncertainty analysis 14

SI Figures 16

Fig. S1 Life cycle assessment system boundary. 16

Fig. S2 Average net GHG balances, average GHG balance breakdown by life cycle stages, and average total carbon stock change breakdown by carbon pools of AR on marginal land in the southeastern U.S. at the end of year 25, 50, 75, and 100. 17

Fig. S3 GHG balance breakdown by life cycle stages of AR on marginal land in the southeastern U.S. over 100 years in different scenarios. 18

Fig. S4 The average life-cycle biogenic carbon balance of S1–S3 in high-density with the thinning case of one rotation (25 years).. 19

Fig. S5 Total carbon stock change breakdown by carbon pools AR on marginal land in the southeastern U.S. over 100 years under different scenarios. 20

Fig. S6 Spatial distributions of (A) site index of loblolly pine base age 25 and (B) marginal land occupation rate per pixel. 21

Fig. S7 The process diagram of biochar plant 22

Fig. S8 Carbon flows of life-cycle stages and carbon pools.. 23

Fig. S9 Total carbon growth curve of loblolly pine plantations in the southern US. 24

Fig. S10 Structure of RothC model. 25

SI Tables 26

Table S1. Parameter ranges used in sensitivity analysis. 26

Table S2. Maximum and minimum input data (average of all pixels) used in uncertainty analysis for forest system. 28

Table S3. Contributions of uncertainties by parameters to the net GHG balances and total carbon stock changes for each scenario. 29

Table S4. Values of key parameters related to biochar end-of-life 30

Table S5. Total carbon stock change (Gt C) under different scenarios by states at the end of year 25, 50, 75, and 100. 31

Table S6. Statistic data of estimated log production of loblolly pine under different scenarios in the southeastern US. 32

Table S7. Spatial datasets used in this study. 33

Table S8. Inputs and assumptions for forest operations. 34

Table S9. The life-cycle GHG emissions of materials and fuels used in this study. 35

Table S10. Ecoinvent 3.6 cut-off database processes used. 36

Table S11. Parameters for lumber production. 37

Table S12. Parameters for CLT production and end-of-life. 38

Table S13. Setting, input parameters, and variables for PMRC model. 39

Table S14. Parameters used to estimate dry mass whole living standings, snags, removed trees from thinning, and litterfall of loblolly pine plantations. 40

Table S15. Parameters for modeling wood waste landfilling. 41

Table S16. Average structural material usage for 1 m^2 floor area. 42

Table S17. Values of key parameters for biochar production 43

SI References 44

SI Note 1: Summary of Methodology

This study presents a cradle-to-grave multi-scale dynamic LCA that integrates a GIS model, a forest growth model, SOC model, and process-based models of manufacturing wood products (i.e., lumber, CLT, and biochar) to quantify the carbon implications of afforestation and reforestation (AR) on the marginal land in the southeastern US over 100 years. The GIS model extracted and processed marginal land layers from a study (1) that identified AR opportunities across the US. The carbon sequestration by forest and biomass carbon was simulated by the forest growth and yield model over 100 years. Then carbon emissions from soil were simulated by the SOC model RothC (2) with the carbon input from the forest (e.g., litterfall, roots, residues, and snags). The GHG emissions by the production of three wood products were simulated by process-based models. This study includes the carbon associated with the end of life (EOL) of wood products as well as the upstream production of fuels, chemicals, and electricity consumed within the system boundary. Scenarios are established to explore the impacts of various forest plantation management strategies and product choices. The functional unit is the whole available marginal land for AR in the southeastern US (2.1 Mha). The Intergovernmental Panel on Climate Change (IPCC) AR6 GWP-100 factors are used to convert GHG emissions to CO_2 equivalent basis (3). Detailed methods are discussed in the sections below (SI Notes 2 to 9).

In this cradle-to-grave LCA, the net GHG balances ($netGHG$) and the total carbon stock (C_{Total}) are calculated by equations (S1) and (S2), respectively. Fig. S8 shows the carbon flows associated with different life-cycle stages and carbon pools.

$$netGHG = (-CO_{2Seq} + GHG_{Forest}^{Bio} + GHG_{Forest}^{Fossil}) + (GHG_{Production}^{Bio} + GHG_{Production}^{Fossil}) + (GHG_{EOL}^{Bio} + GHG_{EOL}^{Fossil}) \quad (S1)$$

$$C_{Total} = (C_{ForestStanding} + C_{SoilForestFloor}) + C_{Product} + C_{EOL} \quad (S2)$$

Where $-CO_{2Seq}$ (estimated by forest growth model) is the accumulative CO_2 sequestration by forest growth, GHG_{Forest}^{Bio} (estimated by RothC model) stands for accumulative CO_2 emissions from the forest that includes the biogenic emissions from biomass decomposition in soil and forest floor, GHG_{Forest}^{Fossil} represent fossil GHG emissions caused by fuels used in forest operation. $GHG_{Production}^{Bio}$ and $GHG_{Production}^{Fossil}$ are biogenic and fossil GHG emissions at the stage of production (producing wood products and biochar), respectively. GHG_{EOL}^{Bio} and GHG_{EOL}^{Fossil} are biogenic and fossil GHG emissions at the end-of-life stage of the products. In equation (S2), $C_{ForestStanding}$ (estimated by forest growth model) is the carbon stock in forest standing, $C_{SoilForestFloor}$ (estimated by RothC model) stands for carbon stock in soil and forest floor, and $C_{Product}$ and C_{EOL} represent carbon stock in products (wood products and biochar) and product end-of-life, respectively. All GHG emissions and carbon stock changes during wood product production and wood product end-of-life along with fossil GHG emissions by forest operation are estimated by the LCA model for wood products manufacturing.

For baseline (protection AR), the net GHG balance ($netGHG_{baseline}$) can be adapted shown as equation (S3) based on equation (S1) because there is no log harvesting for wood product manufacturing (or say $GHG_{Production}^{Bio}$, $GHG_{Production}^{Fossil}$, GHG_{EOL}^{Bio}, GHG_{EOL}^{Fossil} , $C_{Product}$, C_{EOL} are zero).

$$netGHG_{baseline} = -CO_{2Seq} + GHG_{Forest}^{Bio} + GHG_{Forest}^{Fossil} \quad (S3)$$

Biogenic carbon mass balances of the forest system (I in Figure S8): inputs = outputs + carbon stock changes. Inputs are carbon uptake (C_{seq}), outputs are logs and residues and carbon emissions from forests (C_{Forest}^{Bio}). The carbon stock changes include changes in carbon stock in standing forests ($\Delta C_{ForestStanding}$) and soil and forest floor ($\Delta C_{SoilForestFloor}$).

$$C_{seq} = C_{logs\&residues} + C_{Forest}^{Bio} + \Delta C_{ForestStanding} + \Delta C_{SoilForestFloor} \qquad (S4)$$

Based on equation (S4) and the assumption that all carbon losses from forest systems are emitted as CO_2, we can derive the equation (S5) for baseline production AR. $C_{logs\&residues}$ are intermediate flows that go to wood product manufacturing, which are zero for the baseline production AR.

$$-\frac{44}{12} \cdot \left(\Delta C_{ForestStanding} + \Delta C_{SoilForestFloor}\right) = -CO_{2Seq} + GHG_{Forest}^{Bio} \qquad (S5)$$

According to equation (S5), the net differences between biogenic GHG emissions and CO_2 sequestration from the forest system ($-CO_{2Seq} + GHG_{Forest}^{Bio}$) can be calculated using the carbon stock changes of standing forests and soil & floors ($-\frac{44}{12} \cdot \left(\Delta C_{ForestStanding} + \Delta C_{SoilForestFloor}\right)$. We use this method (S5) and (S3) to calculate the net GHG balances of the baseline protection AR because GHG_{Forest}^{Bio} cannot be directly estimated for a 100 year time frame – the forest growth model has a maximum modelling period of 35 years, without the annual carbon input from year 36 to 100, RothC cannot simulate GHG emissions from the soil and forest floor. The calculation of carbon stock change in the forest standing $\Delta C_{ForestStanding}$ are documented in S1 Note 2 and carbon stock change in soil and forest floor $\Delta C_{SoilForestFloor}$ are documented in SI Note 3.

SI Note 2. Forest growth and yield

In this study, we used the 1996 Plantation Management Research Cooperative (PMRC) whole stand growth and yield model to simulate forest growth for site-prepared loblolly pine (*Pinus taeda* L.) plantations across the three prominent physiographic regions in the southern US: piedmont, upper coastal plain, and lower coastal plain in the southeastern US (4). The data for establishing the models come from hundreds of permeant plots in Alabama, Georgia, North Carolina, South Carolina, and Virginia, the first of which was established in 1977. Those plots were carefully established across a wide geographic range and re-measured throughout the rotation of the stand. Each individual tree in the permanent plot was measured and those were compiled into stand-level estimates of dominant height, basal area, and tree density. Those stand-level estimates were used to build the 1996 PMRC whole stand models used in our analysis. The model consists of equations to predict the whole stand: 1) dominant height/site index, 2) survival rate as trees per acre, 3) basal area per acre, 4) yield per acre, and 5) yield breakdown. Detailed equations can be found in the PMRC Technical Report (4). In addition to the whole stand estimation model, thinned plantation and the effects of mid-rotation fertilization with N and P are considered for the growth and yield simulation in the PMRC model. Thinned basal area is estimated as a function of the number of trees thinned. A thinned growth response in terms of per-acre basal area is formulated by comparing the basal area growth of a thinned plantation to that of an un-thinned counterpart of the same age, dominant height, and number of trees per acre. The effect of fertilization is estimated by accounting for a growth response in the dominant height and per-acre basal area growth equations. The growth response is estimated as a function of pounds of elemental nitrogen per acre, whether Phosphorus was applied, and the number of years since treatment.

The main input variable for the model under each scenario is site index base age 25 for loblolly pine with a range from 50 to 105 feet**.** The GIS data of site index for loblolly pine was extracted from the Gridded Soil Survey Geographic (gSSURGO) Database (5). Key variables that differ by scenario include: planning horizon (years), stand density (trees/acre), whether and when thinning is included, and density removed by thinning. Other common variables are whether fertilization is included, fertilization age, and fertilization application rate. The settings, input parameters, and variables for the model in this study are shown in Table S13. For commercial plantations, standings were simulated by PMRC model for 25 years as a rotation with 4 rotations in total. For protection AR, standings were first simulated by PMRC model for 35 years which is the longest timescale that the PMRC can simulate. Then the tree growth and yield starting from year 36 to 100 were estimated by multiplying the estimated total carbon in year 35 from PMRC model by annual growth rates from year 35 to 100. The annual growth rates were calculated from a 100-year tree growth curve (Fig. S9) using a polynomial equation of loblolly pine in the southern US. This growth curve was estimated using a dataset extracted from the US Department of Agriculture Forest Service EVALIDator 2.0.3 online database (6). This dataset consists of 100-year total carbon (except for soil organic carbon) of loblolly pine plantations with a 5-year interval in the southern US, including Alabama, Georgia, Mississippi, North Carolina, South Carolina, Florida, Tennessee, and Virginia states.

The annual total dry mass of the whole living stand, snags, and removed trees from thinning of loblolly pine for a specific site index was estimated using equations (S6–S8) from literature where

the dry mass metric ton of foliage per ha (F, Mg/ha), branch (BR, Mg/ha), stem (S, Mg/ha), and root (Mg/ha) can be calculated with modeled diameter outside-bark at breast height 1.37m (4.5 ft) (dbh, cm), total height (H, m), age (AGE), diameter distribution, and annual standing density that is generated from PMRC 1996 model (7). Other outputs from PMRC model include basal area (BA, m^2/ha) and quadratic mean diameter (Dq, cm). A dbh to Dq ratio of 1.04 based on the literature (7) was used to calculate the dbh.

$$F = a_1 \cdot (dbh^{a_2}) \cdot (e^{a_3 \cdot dbh}) \cdot (H^{a_4}) \cdot (AGE^{a_5}) \cdot N/1000 \qquad (S6)$$

$$BR = b_1 \cdot (dbh^{b_2}) \cdot (e^{b_3 \cdot dbh}) \cdot (H^{b_4}) \cdot (AGE^{b_5}) \cdot N/1000 \qquad (S7)$$

$$S = c_1 \cdot (dbh^{c_2}) \cdot (H^{c_3}) \cdot (AGE^{c_4}) \cdot N/1000 \qquad (S8)$$

Where N represents living tree density or numbers of snags and thinned trees each year (number of trees per ha), 1000 is unit conversion factor from metric ton to kg, a_n, b_n, and c_m (n is from 1 to 5 and m is from 1 to 4) are parameters used in equations (S6–S8) for loblolly pine and are listed in Table S14 (7).

The annual dry mass of litterfall biomass was calculated based on the needlefall (NF, Mg/ha) to litterfall (LF, Mg/ha) ratio (NF/LF) that was calculated by equation (S9) from literature (8). Annual day mass needlefall biomass was estimated by equations (S10–S15) from literature (8). Root mass (Mg/ha) is assumed to be 50% of stem mass, according to literature (9).

$$NF/LF = (d_1 \cdot e_1 + f_1 \cdot AGE^{g_1})/(e_1 + AGE^{g_1}) \qquad (S9)$$

$$LAI = \beta_0/(1 + e^{-((SDI-\beta_2)/\beta_1)}) \qquad (S10)$$

$$SDI = N \cdot [(200 \cdot \sqrt{BA/N \cdot \pi})/25.4]^{1.6} \qquad (S11)$$

$$\beta_0 = d_2 + e_2 \cdot SI \qquad (S12)$$

$$\beta_1 = d_3 + e_3 \cdot SI \qquad (S13)$$

$$\beta_2 = d_4/[1 + (SI/e_4)]^{f_4} \qquad (S14)$$

$$NF = e^{(d_5 + e_5 \cdot \ln(LAI))} \qquad (S15)$$

Where LAI is leaf area index, SDI is stand density index in metric unit, N represents living stand density (number of trees per ha), SI is site index (m), d_n, e_n, f_1, f_4, and g_1 (n is from 1 to 5) are parameters used in equations (S9–S15) for loblolly pine and are listed in Table S14 (8).

SI Note 3. Forest SOC modeling

RothC model uses five conceptual carbon pools to simulate SOC turnover, including four active pools (Resistant Plant Material (RPM), Decomposable Plant Material (DPM), Microbial Biomass (BIO), and Humified Organic Matter (HUM)) and one inactive pool (Inert Organic Matter (IOM)) (Fig. S10) (2). As shown in Fig. S10, carbon from plant materials is divided into DPM and RPM based on a material-specific DPM/RMP ratio. Then CO_2, BIO, and HUM are formed from the decomposition of DPM and RPM according to CO_2/(BIO+HUM) ratio and BIO/HUM ratio. The CO_2/(BIO+HUM) ratio relies on the soil clay content, while the BIO/HUM ratio is 0.85 (2). More CO_2, BIO, and HUM are formed from BIO and HUM. The equations for calculating the amount of organic carbon in a carbon pool that decomposes in a particular month can be found in the model user document (2). RothC can be run with two modes: forward and inverse. For the forward modes, changes in SOC are calculated for equilibrium state or short-term with known monthly plant carbon input and other input data, including monthly precipitation, monthly open pan evaporation, monthly mean air temperature, clay content of soil, DMP/RPM ratio, soil cover state, monthly manure input (if any), and depth of soil layer. For the inverse modes, the model can generate monthly plant carbon input at an equilibrium state with known total SOC content and other input data discussed above (2).

This study simulated SOC changes for a century timescale with initial SOC contents of the five carbon pools and known monthly carbon input from tree materials that was calculated by the forest growth and yield model. However, the data of five carbon pools are rarely known from existing soil databases. To build the initial SOC contents for the five carbon pools, we first calculated the quantity of plant carbon monthly required by soil to maintain an equilibrium state using the inverse mode of RothC and present total SOC content data collected from existing soil databases. In year zero of the study timeframe, the SOC is assumed to be at an equilibrium state. The results of monthly plant carbon input generated from this step were then used to estimate the initial SOC contents for the five carbon pools in year zero using RothC in forward mode. In step three, these modelled initial SOC content of the five carbon pools were used to run a short-term run (100 years) in the forward mode to simulate changes in total SOC content. Varied monthly plant carbon input from forest litterfall, biomass from thinning, snags, harvesting residues, and post-harvest roots under different scenarios were calculated by the forest growth and yield models discussed in SI Note 2 and used as inputs to run the model in step three. A DPM/RPM ratio of 0.25 is used for woodland (2). Other assumptions include: 1) 30 cm topsoil depth, 2) all year-around soil covered by trees, 3) no manure input.

For protection AR (baseline), we implemented an average value of 0.23 Mg C/ha/yr for annual soil carbon stock increase for baselines (10). For the calculation of GHG emissions from soil and forest floor for baseline, detailed calculations can be found in SI note 1.

SI Note 4. Lumber Production

The first unit operation in saw mills is debarking (11). Then logs without bark are sawn into green lumber, slabs/chips, and wet sawdust. The yield of wet lumber, slabs/chips, and wet sawdust from logs was estimated based on the literature data (12, 13) and documented in Table S11. In this study, all the slabs/chips were assumed to be sold to produce durable wood products (12). Bark and wet sawdust, as well as dry shavings/chips and sawdust from the planing (planing process is discussed as follows), were used for energy generation in the saw mill.

Wet lumber are dried at 90–120℃ (dry bulb temperature) in a dry kiln operating to reach the targeted moisture content (14, 15). Mill residues (bark, wet sawdust, dry planing shavings/chips and sawdust) are combusted to provide the energy for the dry kiln. When mill residues are not sufficient for the energy demand, natural gas is used; when energy is excessive, mill residues are used for power generation (12).

The energy demand for kiln drying was determined as the total heat demand in drying divided by the overall energy efficiency for energy generation and drying. The data were collected from the literature and shown in Table S11 (14, 16, 17). The total heat demand was determined based on the the desorption heat (MJ) needed for evaporating 1 kg water out of wood (18). The total energy demand for kiln drying is met by the total lower heating value (*LHV*) of mill residues, given by equation (S16) (19, 20). *HHV* is higher heating value (assumed 20 MJ/oven dry kg (odkg) for wood and 20.5 MJ/odkg for bark) (12); *MC* is moisture content (*dry basis*) of mill residues; *H* is hydrogen content percentage in fuel (assuming 6) (16, 19–21). The LHV for natural gas is 47.1 MJ/kg according to (22).

$$LHV = HHV - 0.0245 \cdot \left(\left(\frac{MC}{1 + MC} \right) \cdot 100 + 9H \right) \quad \text{(S16)}$$

Then the dried lumber is planed to remove uneven surface and generate finished lumber (23). Dry shavings/chips and sawdust generated in this process are viewed as mill residues (16, 23). Then dimensional lumber is ready for distribution to the market.

As a structural material, the life span of lumber is assumed as 30 years based on the report by the National Association of Home Builders and Bank of America Home Equity (24). Then lumber is assumed to be landfilled. The calculation of end-of-life GHG emissions is shown in SI Note 5.

SI Note 5. Landfill of wood waste

Landfilled wood waste emits GHG emissions (majorly CO_2 and CH_4) through a decay process (25–27). Due to the high GWP factor of CH_4 (27.0 for non-fossil CH_4 GWP-100) and potential energy recovery value of CH_4, landfill gas recovery for energy generation is in trend in the US according to the US Environmental Protection Agency (EPA) (28). In this study, the GHG emissions of landfill are estimated in two steps. First, CH_4-rich GHG emissions from landfill decay were estimated based on the Intergovernmental Panel on Climate Change (IPCC) First Order Decay method (12, 25). Second, part of the landfill gas is recovered and combusted for power generation. All the parameters and values are collected from the literature and documented in Table S15. The GHG emissions from landfilled lumber and CLT are estimated using the same methods presented in this section.

Equations S17 and S18 show the method of the IPCC First Order Decay (25).

$$C_{decomposed} = W \cdot DOC \cdot DOC_f \cdot \left(1 - e^{-kt}\right) \qquad (S17)$$

$$CH_{4\,generated} = [(C_{decomposed} \cdot MCF \cdot F \cdot 16/12)(1 - R)] \cdot (1 - OX) \qquad (S18)$$

In equation S17, $C_{decomposed}$ is the accumulative decomposed carbon mass from year 0 to year t. W is the mass of deposited wood waste (*wet basis*); DOC is the degradable organic carbon of wood waste; DOC_f is the faction of DOC that can decompose; k is the landfill decay rate (29). In equation S18, MCF is the CH_4 correction factor which is determined by site management (e.g., disposal depth in the soil, anaerobic conditions) (25); F is the volume fraction of CH_4 in landfill gas; R is the total recovered CH_4 portion by energy recover device (29). This study adopts the typical recovery value of R to be 0.75 based on Anshassi et al. (30). OX is the average oxidation factor describing the fraction of oxidized methane (29). After the CH_4 emission is determined, this study estimated the CO_2 emissions of wood waste landfills using the experimental data on the volume rate of CH_4 (before being recovered) to CO_2 (31). The recovered landfill gas is combusted to generate power. The generated power is calculated based on the total LHV of landfill gas and electricity generation efficiency (30). Then the substitution credit for the generated power is considered to replace the US SERC region market mix using the data from ecoinvent 3.6 cut-off database (32). The detailed values are shown in Table S15.

SI Note 6. CLT production and potential substitution benefits

At the CLT plant, lumber preparation ensures the quality of CLT production, including grading, grouping, and moisture content screening (33–35). In lumber preparation, the boards undergo visual grading and grouping process. The preparation groups the lumber for different layers and directions, since the lumber in longitudinal layers need to reach visual grade No. 2 and the lumber in transverse layers need to reach visual grade No. 3 (36). Then the lumber moisture content is measured. In this study, the dimensional lumber from the saw mills has reached the target moisture of 12%, therefore additional drying is not necessary. Then the selected and grouped boards are longitudinally end-jointed to make long, continuous lumber for layering and gluing (40). Finger-jointing is assumed to be the end-jointing type (12, 37). The longitudinally assembled lumber is then layered, glued with resin, and pressed to form CLT panels (38). The resin in this study was selected to be melamine formaldehyde (MF), a commonly adopted resin in finger-jointing and face-bonding (39, 40). After pressing, the finishing steps are planing and end cutting. Planing can remove excessive resin and finalize uneven surfaces. End cutting is finished by Computerized Numerical Control (CNC) to reach the customized shape (41). CLT is then packaged and ready for transport to the construction site. The waste produced in the CLT plant are landfilled. The data (e.g., energy and chemical usage) for this stage were collected from the literature and documented in Table S12.

This study includes the potential substitution benefits of CLT replacing traditional reinforced concrete and steel as building materials (42). The structural material consumption for CLT buildings and steel & concrete buildings were estimated based on the work by D'Amico et al. (42). Table S16 shows the average structural material usage for two types of buildings per m2 floor area (42, 43). Then the processes from ecoinvent database (see Table S10) were used to determine the GHG emission reduction per m^2 floor area, and the results were normalized to 1 metric ton CLT panel used (1.13 t CO_2e t^{-1} CLT panel used). For steel, after the usage, the end-of-life recycling rate is assumed to be 35% based on the ecoinvent 3.6 cut-off database (43). The remaining steel is landfilled. The upstream production burdens of steel and concrete products and landfill of steel were derived from ecoinvent 3.6 cut-off database (see Table S10) (32).

SI Note 7. Biochar Production

The flowchart of the biochar plant is shown in Fig. S7. In pretreatment, initial grinding reduces the feedstock size to ~50 mm (44). Then the feedstocks are dried in the rotary drum dryer to reach the moisture content 10% (*green weight basis*) in an ambient temperature range of 160 ℃ to 180 ℃ (45). The dried biomass is further ground in the hammer mill to reach 2.5–3.8 mm. Biomass is then pyrolyzed in a fluidized bed reactor at 500℃ and 1 atmosphere for 60 minutes in the nitrogen ambient (46). Pyrolysis outputs include biochar and gaseous products. Biochar is separated from the gaseous products that are ducted to the combustor to provide heat for pretreatment and pyrolysis. Pyrolysis kinetics were simulated in Aspen Plus through the multistep reaction mechanism method (MSRM) (46, 47). In the simulation, the biomass feedstock is first decomposed into major lignocellulosic components (cellulose, hemicellulose, and lignin) and then goes through a series of reactions (48). This study selects glucose for cellulose, xylose for hemicellulose, lignin-C, lignin-O, and lignin-H for lignin (48). As the biomass composition data were collected from the ultimate analysis (e.g., carbon content, oxygen content), the triangular method was applied to calculate the mass fraction of each model compound (49). In total, four reactors were used in the simulation. This first reactor (RYield) breaks the biomass into five model compounds and ash, the second reactor (RBatch) simulates the primary pyrolysis kinetic reactions, the third reactor (RCSTR) simulates the tar cracking reactions, the fourth reactor turns the remaining metaplastic components into biochar (50).

Then multi-stage cyclones separate the biochar from the gaseous product. The gaseous products from the pyrolyzer are combusted to produce heat for the rotary drum dryer and pyrolysis reactor. If the heat from the gaseous product is not sufficient, natural gas will be combusted. The pyrolysis reaction parameter settings are provided in Table S17. The electricity and diesel consumption of unit operations in the biochar plant and the other inputs are displayed in Table S17.

SI Note 8. End-of-life of biochar

This study used the three-pool exponential decay method presented by Woolf et al. (51) to model the GHG emissions from the slow decay of biochar after application, as shown in equation (S19). c_{remain} is the remaining carbon mass of biochar after year t for the initial carbon input $c_{initial}$ (51). $C_{initial}$ is derived from the Aspen Plus simulation. Three carbon pools in biochar are C_1, C_2, C_3, respectively, with corresponding decay rate k_1, k_2, k_3. The value of C_1, C_2, C_3, k_1, k_2, k_3 are derived from the regression data of experimental results (51, 52) and displayed in Table S4. F_T is the ratio of target temperature T to the reference temperature T_{ref} and is a function of Q_{10} as shown in equation (S20). T_{ref} in this study adopted the literature data of biochar decay experimental results (pine wood-derived biochar) (52) (see Table S4). T is the annual average temperature of the area where biochar is applied. As mentioned in the main text, this study assumes that derived biochar is applied in similar climate conditions with forest locations. Hence, T adopts the same annual average temperature of forest locations. Q_{10} describes the impacts of temperature on the decomposition rates of biochar carbon (51) and is derived by equation (S21).

For the whole area studied in this work, averagely, the mean residence time (MRT = $1/k$) of the biochar is 550 years (53, 54). However, the MRT of the topsoil SOC in the forests highly depends on climate conditions such as temperature and precipitation. MRT of SOC is generally lower in low-latitude zones and increasing toward high-latitude zones. One estimated range of mean MRT of soil organic carbon in the study area (N30°–N40°) is 14–16 years (55), another indicates a range of 12–29 years for temperate forests (56), and some others show a range of 10–30 years for the southeastern US (57, 58), which are all much shorter than the MRT of biochar. If forest residues are left in soil to form SOC instead of being converted to biochar, it is expected that the biogenic carbon stored in SOC will resident much shorter time than the carbon stored in biochar.

$$c_{remaining} = c_{initial} \times \left(C_1 \times e^{(-k_1 \times F_T \times t)} + C_2 \times e^{(-k_2 \times F_T \times t)} + C_3 \times e^{(-k_3 \times F_T \times t)}\right) \quad (S19)$$

$$F_T = e^{\left(\ln(Q_{10}) \times \frac{T-T_{ref}}{10}\right)} \quad (S20)$$

$$Q_{10} = (1.1 - 63.1579 \times \frac{e^{(-0.19T)} - e^{\left(-0.19T_{ref}\right)}}{T-T_{ref}} \quad (S21)$$

SI Note 9. Uncertainty analysis

Uncertainty analysis of this study is addressed by the equations S22–S23 below.

$$Total_{pess} = Model\left(Forest_{pess}\ , Product_{pess}\right) \qquad (S22)$$

$$Total_{opt} = Model\left(Forest_{opt}, Product_{opt}\right) \qquad (S23)$$

$Total_{pess}$ and $Total_{opt}$ are the pessimistic and optimistic values of the results (i.e., net carbon stock and net GHG balances) that are impacted by two system, namely forest system and product system. $Forest_{pess}$ and $Forest_{opt}$ are the pessimistic and optimistic carbon benefits of the forest system; $Product_{pess}$ and $Product_{opt}$ are pessimistic and optimistic values of product-level gate-to-grave (including production and end-of-life) GHG emissions.

$$Forest_{pess} = Model(ForestCY_{min}, SOC_{min}) \qquad (S24)$$

$$Forest_{opt} = Model(ForestCY_{max}, SOC_{max}) \qquad (S25)$$

$Forest_{pess}$ and $Forest_{opt}$ are derived from equations (S24) and (S25). $ForestCY_{min}$ and $ForestCY_{max}$ are the minimum and maximum values of forest carbon yield (Mg C/ha) that describes the carbon yield in harvested logs, residues, litterfall, and dead roots. $ForestCY$ is determined by equation (S26) as shown below. $ForestBiomassYield$ describes the biomass yield from the forest and generated based on the methods described in SI Note 2. GUF is the forest growth uncertainty factor (85%–115%) (see Tables S2 and S13) (59). In other words, the uncertainty range of the modeled forest growth is ±15% (see Tables S2 and S13) (59). $CarbonContent$ is the carbon content (as a percentage) of loblolly pine that is estimated at the 95% confidence interval based on literature data (60–76) (see SI Tables S1 and S2).

$$ForestCY = ForestBiomassYield \times GUF \times CarbonContent \qquad (S26)$$

In equations (S24) and (S25), SOC_{min} and SOC_{max} are the minimum and maximum value for the simulated SOC stocks from RothC, respectively. For SOC_{min} and SOC_{max}, the minimum and maximum SOC in uncertainty analysis is calculated by equations (S27)–(S28) derived from the method by the Food and Agriculture Organization (FAO) of the United Nations (59).

$$SOC_{min} = Model(SOC_{Initial\,min}, ForestCY_{min}, Temp_{max}, Pre_{max}, Clay_{min}) \qquad (S27)$$

$$SOC_{max} = Model(SOC_{Initial\,max}, ForestCY_{max}, Temp_{min}, Pre_{min}, Clay_{max}) \qquad (S28)$$

Where $SOC_{Initial\,min}$ and $SOC_{Initial\,max}$ are repectively the minimum and maximum value of the initial SOC stocks estimated at the 95% confidence interval based on the variation within the aggregated 1km×1km grid cells (considering the original values from 30m×30m grids in the ISRIC-World Soil Information); $ForestCY_{min}$ and $ForestCY_{max}$ are the sources of forest carbon inputs to the soil, including carbons from litterfall, biomass from thinning, snags, harvesting residues, and post-harvest roots; $Temp_{min}$ and $Temp_{max}$ are respectively the minimum and maximum value of monthly mean temperature estimated at the 95% confidence interval based on the interannual variation between year 1991–2020 from the CRU TS 4.05 dataset. Pre_{min} and Pre_{max} are respectively the minimum and maximum value of monthly precipitation estimated at the 95% confidence interval based on the interannual variation between year 1991–2020 from the CRU TS

4.05 dataset. $Clay_{min}$ and $Clay_{max}$ are respectively the minimum and maximum value of soil clay content estimated at the 95% confidence interval based on the variation within the aggregated 1km×1km grid cells (considering the original values from 30m×30m grids in the ISRIC-World Soil Information).

For $Product_{pess}$ and $Product_{opt}$, as stated in the main text, this study deploys the two-step uncertainty analysis for LCA for lumber, CLT, and biochar. The first step is to conduct the sensitivity analysis for the life-cycle GHG emissions for each individual product (i.e., lumber, CLT, and biochar) (the parameters are shown in SI Table S8, S11, S12, S15, S16, and S17). Then those parameters with impacts larger than 5% are selected. The ranges of these parameters used in LCA for lumber, CLT, and biochar, are collected from the literature and shown in SI Table S1. After the sensitivity analysis, there are four parameters selected, namely mill waste recovery rate ($MillRecov$), landfill decay rate (k), landfill MCF (MCF), and potential material substitution for steel frame in traditional buildings by CLT ($Steel$). The second step is to perform the uncertainty analysis for $Product_{pess}$ and $Product_{opt}$ based on equations (S29)–(S30).

$$Product_{pess} = Model(\, MillRecov_{min}, k_{max}, MCF_{max}, Steel_{min}) \qquad (S29)$$

$$Product_{opt} = Model(\, MillRecov_{max}, k_{min}, MCF_{min}, Steel_{max}) \qquad (S30)$$

After deriving $Forest_{pess}$, $Product_{pess}$, $Forest_{opt}$, $Product_{opt}$, the uncertainty analysis is performed based on equations (S22)–(S23).

SI Figures

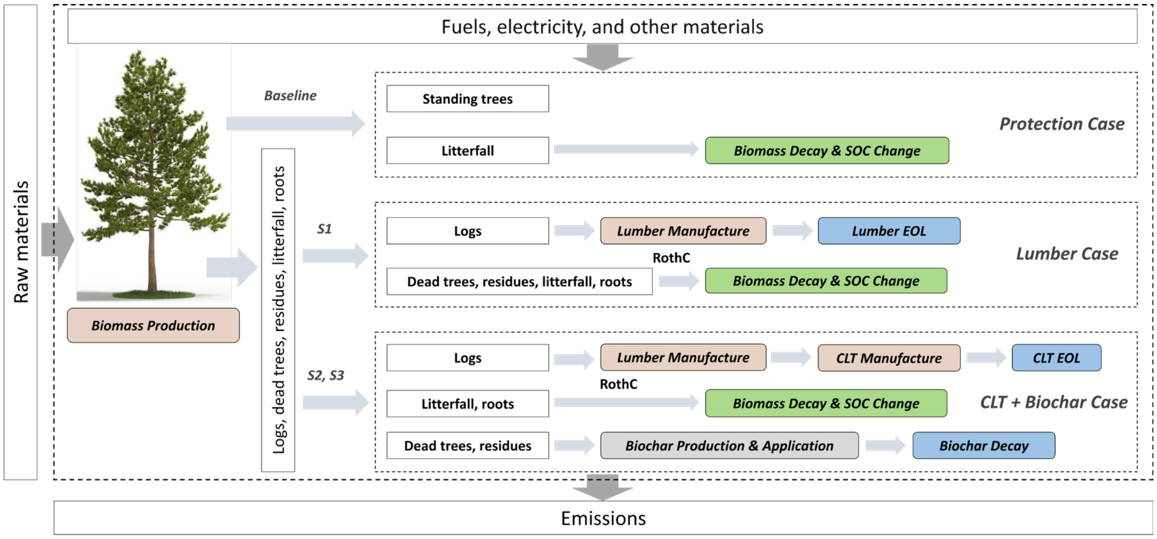

Fig. S1 Life cycle assessment system boundary.

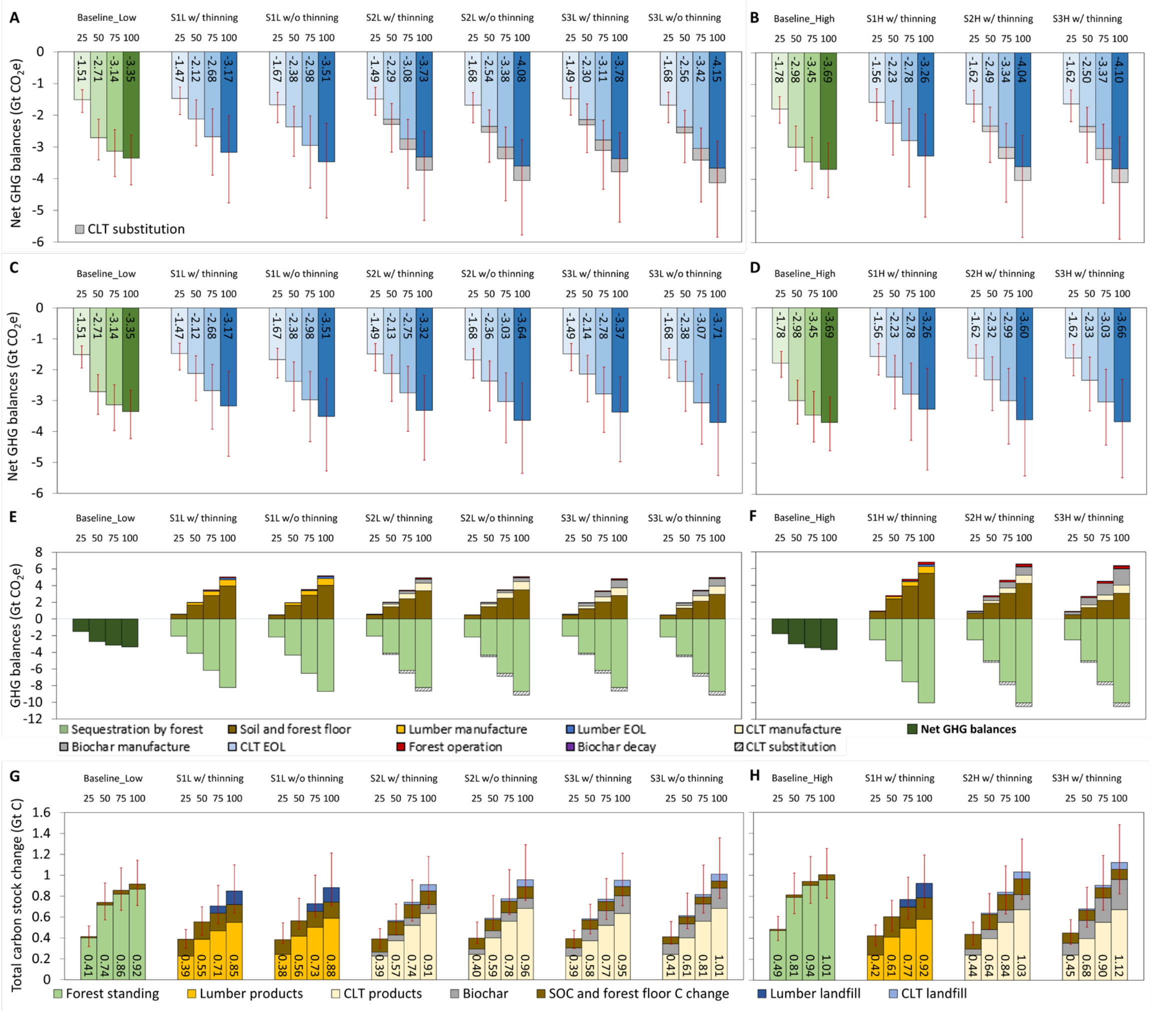

Fig. S2 Average net GHG balances, average GHG balance breakdown by life cycle stages, and average total carbon stock change breakdown by carbon pools of AR on marginal land in the southeastern U.S. at the end of year 25, 50, 75, and 100.

Net GHG balances under (A) low-density scenarios with steel substitution credit, (B) high-density scenarios with steel substitution credit, (C) low-density scenarios with steel substitution credit, (D) high-density scenarios with steel substitution credit. GHG balance breakdown by life cycle stages under (E) low-density scenarios, (F) high-density scenarios. Total carbon stock change breakdown by carbon pools under (G) low-density scenarios, (H) high-density scenarios. Note: the GHG emissions of CLT EOL, Biochar decay, and Forest operation in figures (E) and (F) are not visible due to their minimal values. The maximum ranges of modeled results from uncertainty analysis are shown by the error bars in red.

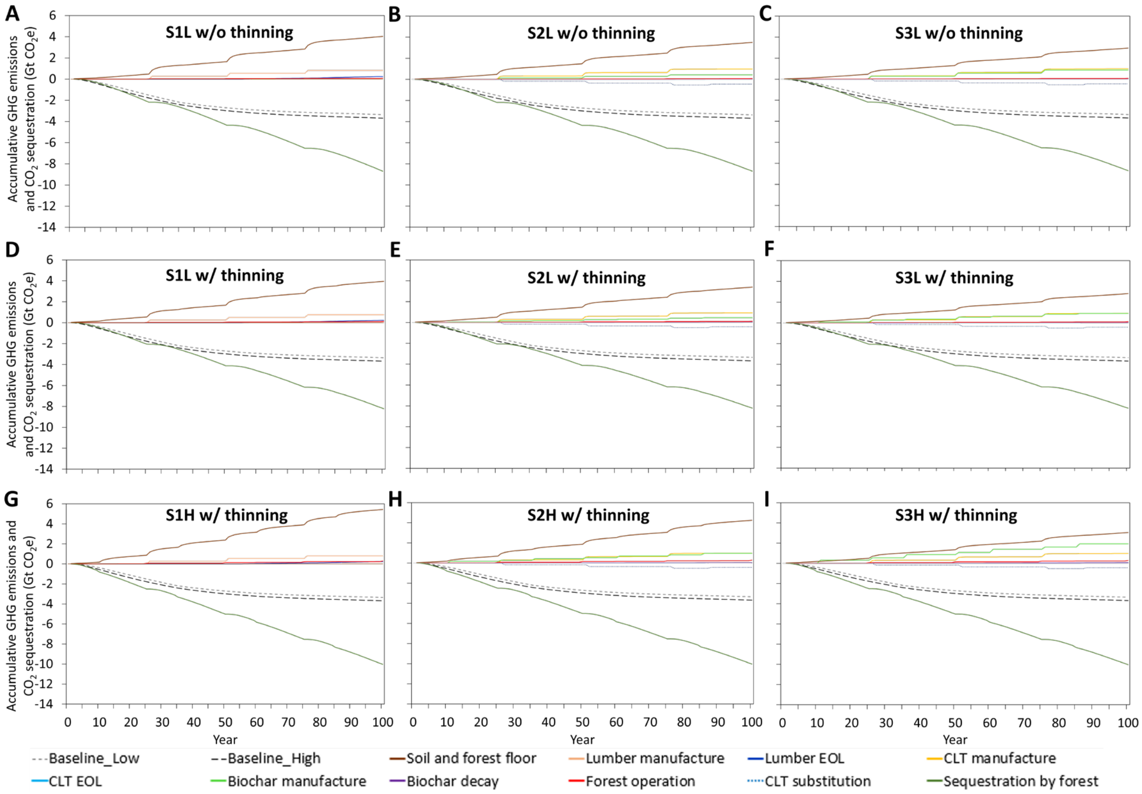

Fig. S3 GHG balance breakdown by life cycle stages of AR on marginal land in the southeastern U.S. over 100 years in different scenarios.

(A) Low density without thinning for S1 — Lumber without forest residues removal, (B) Low density without thinning for S2 — CLT with 50% forest residues removal for biochar, (C) Low density without thinning for S3 — CLT with 100% forest residues removal for biochar, (D) Low density with thinning for S1 — Lumber without forest residues removal, (E) Low density with thinning for S2 — CLT with 50% forest residues removal for biochar, (F) Low density with thinning for S3 — CLT with 100% forest residues removal for biochar, (G) High density with thinning for S1 — Lumber without forest residues removal, (H) High density with thinning for S2 — CLT with 50% forest residues removal for biochar, and (I) High density with thinning for S3 — CLT with 100% forest residues removal for biochar. Note: the GHG emission of CLT EOL and Biochar decay are not visible due to their minimal values compared to others. These figures are based on average values.

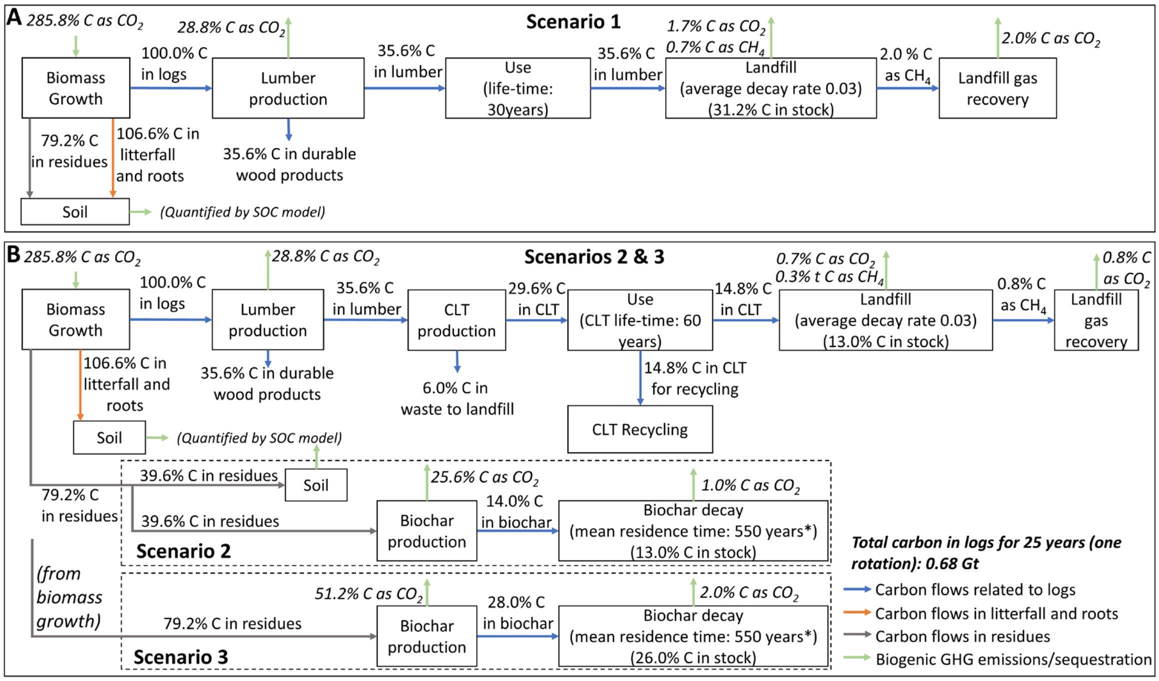

Fig. S4 The average life-cycle biogenic carbon balance of S1–S3 in high-density with the thinning case of one rotation (25 years). (A) S1 — Lumber without forest residue removal, (B) S2 — CLT with 50% forest residue removal for biochar and S3 — CLT with 100% forest residue removal for biochar. Since the end-of-life decay process of landfill waste and biochar is dynamic in this study, the figure shows the carbon balance under averaged decay time with averaged decay rate (lumber 16 years, CLT 15 years, biochar 38 years). The GHG emissions from soil are quantified through the soil organic carbon model RothC (see Materials and Methods).

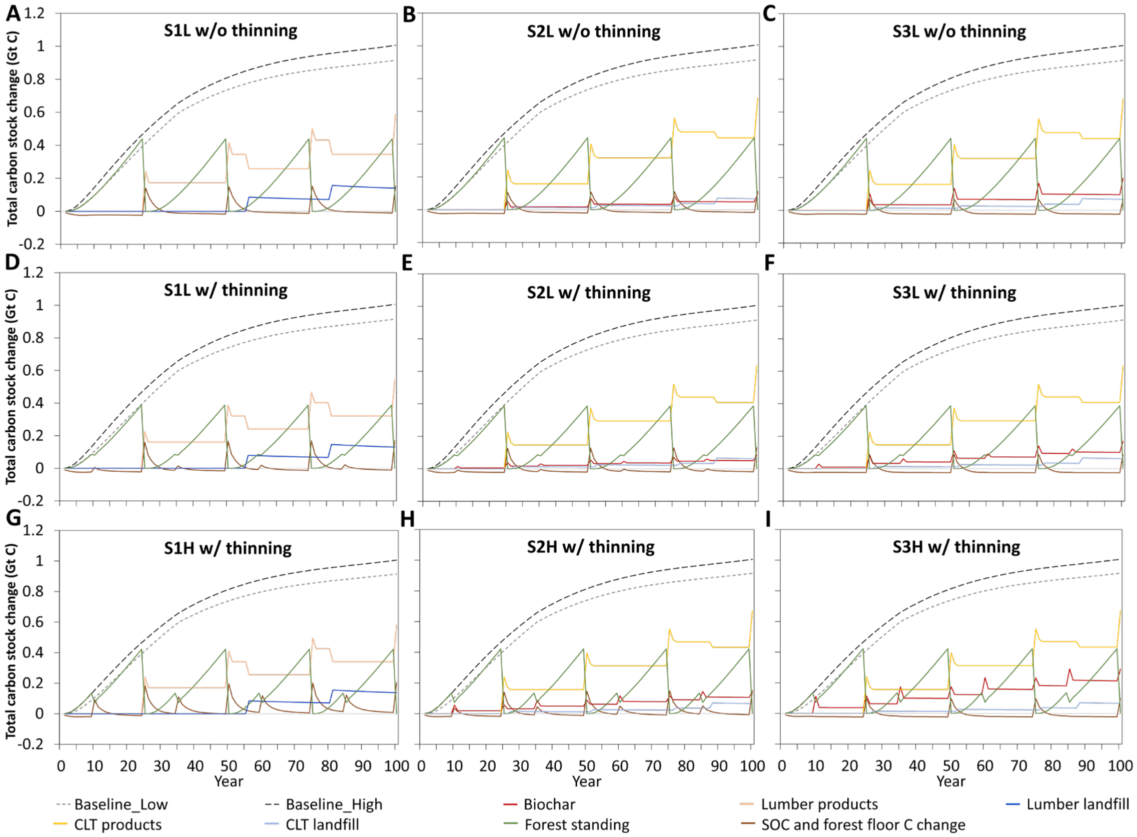

Fig. S5 Total carbon stock change breakdown by carbon pools AR on marginal land in the southeastern U.S. over 100 years under different scenarios.

(A) Low density without thinning for S1 — Lumber without forest residues removal, (B) Low density without thinning for S2 — CLT with 50% forest residues removal for biochar, (C) Low density without thinning for S3 — CLT with 100% forest residues removal for biochar, (D) Low density with thinning for S1 — Lumber without forest residues removal, (E) Low density with thinning for S2 — CLT with 50% forest residues removal for biochar, (F) Low density with thinning for S3 — CLT with 100% forest residues removal for biochar, (G) High density with thinning for S1 — Lumber without forest residues removal, (H) High density with thinning for S2 — CLT with 50% forest residues removal for biochar, and (I) High density with thinning for S3 — CLT with 100% forest residues removal for biochar. These figures are based on average values.

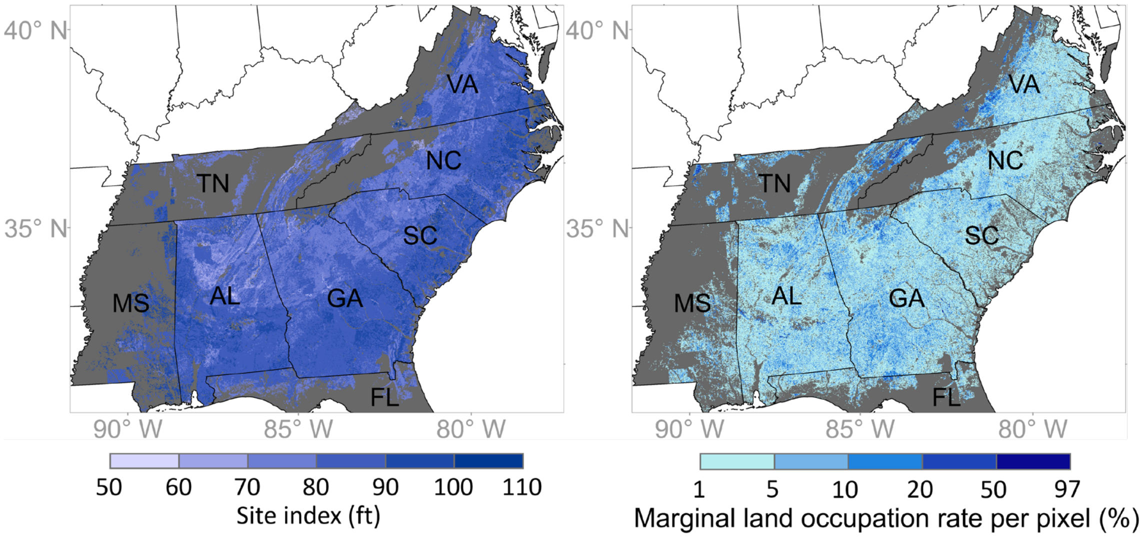

Fig. S6 Spatial distributions of (A) site index of loblolly pine base age 25 and (B) marginal land occupation rate per pixel.

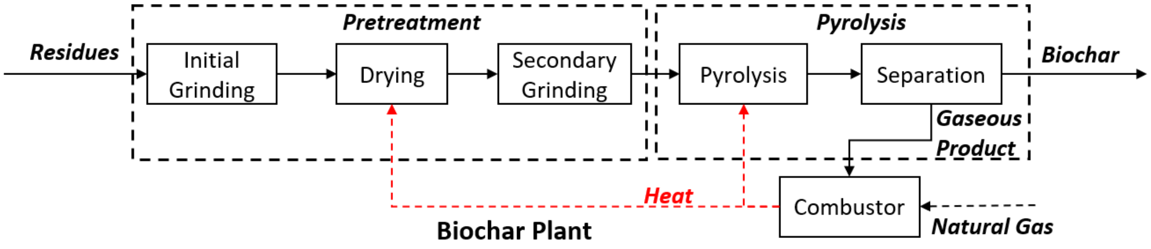

Fig. S7 The process diagram of biochar plant.

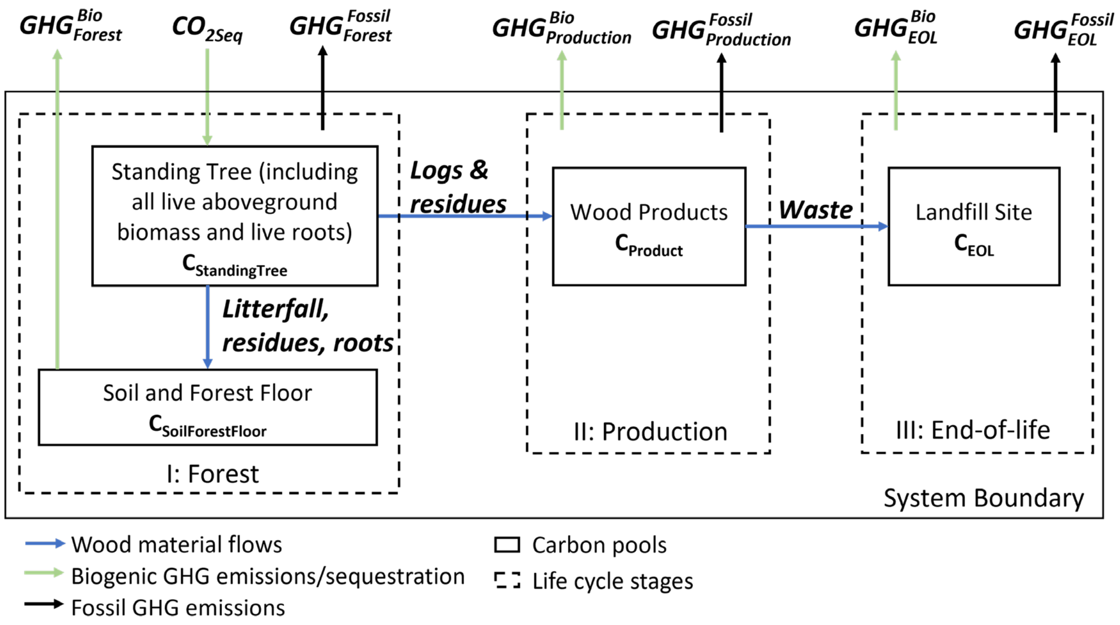

Fig. S8 Carbon flows of life-cycle stages and carbon pools. I: forest system, II: production system of wood products, III: end of life of wood products.

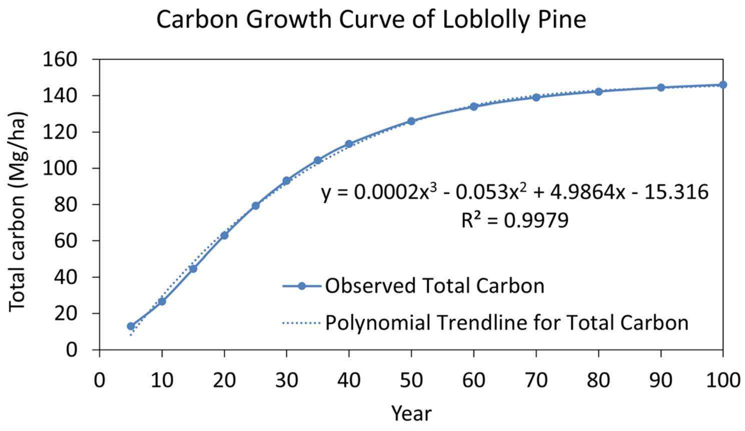

Fig. S9 Total carbon growth curve of loblolly pine plantations in the southern US. Variable x is the age of loblolly pine.

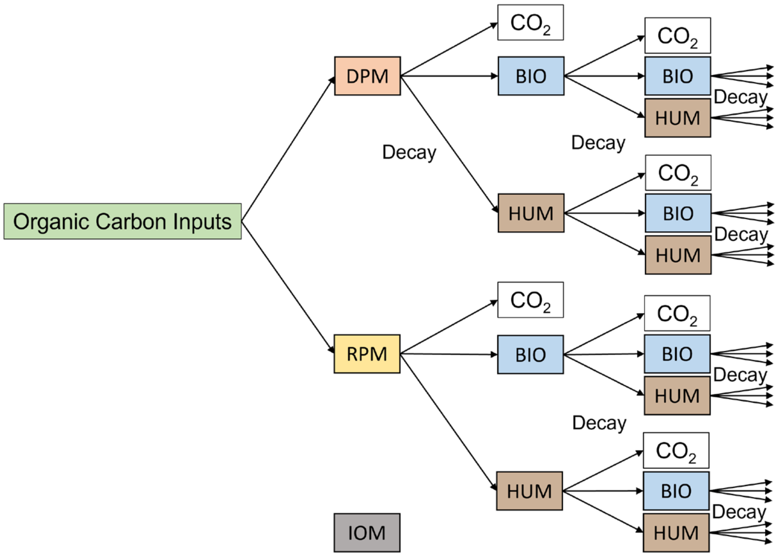

Fig. S10 Structure of RothC model. RPM: Resistant Plant Material, DMP: Decomposable Plant Material, BIO: Microbial Biomass, HUM: Humified Organic Matter, IOM: Inert Organic Matter (2). Both BIO and HUM decompose to form more CO_2, BIO, and HUM. (2)

SI Tables

Table S1. Parameter ranges used in the sensitivity analysis.

Parameter	Unit	Lower limit	Mean value	Higher limit
Forest operations				
Diesel consumption of site preparation and planting (77–81)	kg ha^{-1}	67.0	95.5	118.0
Diesel consumption of applying fertilizers and herbicides (80)	kg ha^{-1} [a]		7.50	
Diesel consumption in logging (12, 78, 80–92)	kg m^{-3}	0.37	1.66	3.57
Diesel consumption of felling (81)	kg m^{-3}		0.31	
Nitrogen fertilizer usage (93)	kg N ha^{-1}	0	103	216
Phosphorus fertilizer usage (93)	kg P_2O_5 ha^{-1}	0	12.8	25.6
Herbicide usage (glyphosate) (80)	kg ha^{-1}	0	1.36	2.72
Live tree carbon content (60–76)	% *dry basis*	45.9	50.0	54.9
Lumber production				
Electricity consumption of sawing (12, 16, 17, 23, 94)	kWh m^{-3} log input	16.5	24.4	32.3
Electricity consumption of kiln drying and kiln heat generation (12–14, 16, 17, 23)	kWh m^{-3} lumber input	17.9	26.9	35.8
Electricity consumption of planing (12, 13, 16, 17, 23)	kWh m^{-3} lumber input	7.7	18.2	28.7
Diesel consumption of hauling materials (12, 60, 95, 96)	kg m^{-3} dried lumber	1.7	3.6	5.5
Gasoline consumption of hauling materials (12, 60, 95, 96)	kg m^{-3} dried lumber	0.03	0.23	0.43
Lumber mill waste recovery (12, 13, 16, 17, 97–107)	%	36.0	50.0	64.0
Lumber target moisture content (12, 13, 15, 34, 37, 100, 101, 106, 108)	% (*dry basis*)	11.0	12.0	14.5
Overall energy efficiency for energy generation and drying (12, 14, 16, 17)	%	16.7	17.9	29.8
Lumber drying shrinkage (12, 100, 101, 109)	%	4.4	9.1	16.0
Planing byproduct mass percentage (12, 13, 16, 17, 110)	%	13.8	17.8	21.8
Average transportation distance from field to lumber mills (16)	km	0	92	184
Average transportation distance of lumber distribution (12)	km	0	213	426
Average hauling distance to landfill site (111–114)	km	0	251	502
Landfilling				
MCF (25, 29, 115, 116)		0.6	0.75	0.9
F (25, 29, 115, 116)		0.475	0.50	0.525
OX (25, 29, 115, 116)		0	0.05	0.1
k (12, 25, 29, 115, 116)		0.02	0.03	0.04
R (30)	%	70%	75%	90%
Power generation efficiency by wood incineration (30)	%	28	30	32
CLT production				

Resin (MF) for finger-jointing and pressing (34, 37, 108, 117)	kg m^{-3} lumber input	5.3	6.1	6.9
Planing shavings percentage (34, 108)	%	3.6	4.0	4.5
End cutting waste percentage (34, 108)	%	12.2	12.8	13.4
Total electricity consumption of CLT production (34, 108, 117, 118)	kWh m^{-3} final CLT produced	98.9	113.8	128.7
Transportation distance from lumber mill to CLT producer (34, 108)	km	91	266	438
Transportation distance from CLT producer to a construction site (12, 34)	km	0	213	426
Transportation distance from construction site to landfill site (111–114)	km	0	251	502
Potential material substitution by using CLT				
Steel usage for 1 m^2 traditional building floor area (42)	kg	38.7	45.1	51.5
Steel usage for 1 m^2 CLT building floor area (42)	kg	22.4	27.7	33.0
CLT for 1 m^2 CLT building floor area (42)	kg	65	70	75
Biochar production				
Diesel consumption in the wheel loader (119)	kg $ODMT^{-1}$ feedstock	0.72	1.4	2.16
Electricity consumption in the grinder (119)	kWh $ODMT^{-1}$ fed in	20	40	60
Electricity consumption in the hammer mill (119)	kWh $ODMT^{-1}$ fed in	16.5	33	49.5
Electricity consumption in the rotary drum dryer (119)	kWh $ODMT^{-1}$ fed in	22.5	45	67.5
Electricity consumption in the feed hopper (119)	kWh $ODMT^{-1}$ fed in	0.85	1.7	2.55
Transportation distance from the forest to the biochar plant (119)	km	0	69.5	139
Transportation distance from the biochar plant to the application field (32)	km	0	118	236

Table S2. Maximum and minimum input data (average of all pixels) used in uncertainty analysis for forest system.

Input data	Unit	Lower limit	Mean value	Higher limit
Forest growth uncertainty factor (*GUF*) [a]	%	85.0	100.0	115.0
Carbon content (*CarbonContent*) [b]	%	45.9	50.0	54.9
Initial SOC content	Mg ha^{-1}	36.47	43.31	56.23
Soil clay content	%	12.44	20.87	30.14
Year-round mean temperature [c]	℃	8.73	10.61	14.32
Year-round total precipitation [c]	mm	272.52	766.16	1475.11

[a] The forest growth uncertainty factor describes the uncertain forest growth relative to the adopted values used in the forest growth model.

[b] See Table S1.

[c] The actual input climate data is monthly data. Here are annual average temperatures and annual total precipitation and evaporation to show the differences in data input between maximum and minimum values.

Table S3. Contributions of uncertainties by parameters to the net GHG balances and total carbon stock changes for each scenario.

Contributions of uncertainties by parameters on net GHG balances (%)

	Baseline Low	Baseline High	S1L w/o thinning	S2L w/o thinning	S3L w/o thinning	S1L w/ thinning	S2L w/ thinning	S3L w/ thinning	S1H w/ thinning	S2H w/ thinning	S3H w/ thinning
LCA modeling parameters in the product system	0	0	12.02	15.40	15.21	12.12	15.36	15.19	11.39	14.21	14.03
Forest system modeling parameters	100	100	87.98	84.6	84.79	87.88	84.64	84.81	88.61	85.79	85.97

Contributions of uncertainties by parameters on carbon stock change (%)

	Baseline Low	Baseline High	S1L w/o thinning	S2L w/o thinning	S3L w/o thinning	S1L w/ thinning	S2L w/ thinning	S3L w/ thinning	S1H w/ thinning	S2H w/ thinning	S3H w/ thinning
LCA modeling parameters in the product system	0	0	8.56	6.92	9.87	8.81	7.85	10.91	8.35	10.88	15.98
Forest system modeling parameters	100	100	91.44	93.08	90.13	91.19	92.15	89.09	91.65	89.12	84.02

Table S4. Values of key parameters related to biochar end-of-life (51, 52).

Parameter	Value
C_1	0.0043
C_2	0.9957
C_3	0
k_1	3.7973
k_2	0.0038
k_3	0
T_{ref}	32.0

Table S5. Total carbon stock change (Gt C) under different scenarios by states at the end of year 25, 50, 75, and 100.

Forest Management	Scenarios	***S1***				***S2***				***S3***			
Low-density without thinning	Year	25	50	75	100	25	50	75	100	25	50	75	100
	VA	0.04	0.07	0.08	0.10	0.04	0.07	0.09	0.11	0.05	0.07	0.09	0.12
	TN	0.01	0.02	0.02	0.03	0.01	0.02	0.02	0.03	0.01	0.02	0.02	0.03
	SC	0.04	0.06	0.08	0.10	0.05	0.07	0.09	0.10	0.05	0.07	0.10	0.11
	NC	0.05	0.07	0.09	0.10	0.05	0.06	0.09	0.11	0.05	0.07	0.09	0.12
	MS	0.01	0.01	0.01	0.01	0.01	0.01	0.01	0.01	0.01	0.01	0.01	0.01
	GA	0.12	0.18	0.24	0.30	0.13	0.20	0.27	0.34	0.13	0.21	0.28	0.34
	AL	0.10	0.14	0.20	0.23	0.10	0.15	0.20	0.25	0.10	0.15	0.21	0.26
	FL	0.01	0.01	0.01	0.01	0.01	0.01	0.01	0.01	0.01	0.01	0.01	0.02
	Sum	0.38	0.56	0.73	0.88	0.40	0.59	0.78	0.96	0.41	0.61	0.81	1.01
	Scenarios	***S1***				***S2***				***S3***			
Low-density with thinning	Year	25	50	75	100	25	50	75	100	25	50	75	100
	VA	0.04	0.06	0.08	0.10	0.04	0.07	0.09	0.10	0.04	0.07	0.09	0.11
	TN	0.01	0.02	0.02	0.03	0.01	0.02	0.02	0.03	0.01	0.02	0.02	0.03
	SC	0.04	0.06	0.08	0.10	0.05	0.07	0.09	0.10	0.05	0.07	0.09	0.10
	NC	0.05	0.07	0.09	0.11	0.05	0.06	0.08	0.11	0.05	0.07	0.09	0.11
	MS	0.01	0.01	0.01	0.01	0.01	0.01	0.01	0.01	0.01	0.01	0.01	0.01
	GA	0.13	0.18	0.24	0.28	0.13	0.19	0.25	0.31	0.13	0.19	0.26	0.32
	AL	0.10	0.14	0.18	0.21	0.09	0.14	0.19	0.24	0.09	0.14	0.20	0.25
	FL	0.01	0.01	0.01	0.01	0.01	0.01	0.01	0.01	0.01	0.01	0.01	0.02
	Sum	0.39	0.55	0.71	0.85	0.39	0.57	0.74	0.91	0.39	0.58	0.77	0.95
	Scenarios	***S1***				***S2***				***S3***			
High-density with thinning	Year	25	50	75	100	25	50	75	100	25	50	75	100
	VA	0.05	0.07	0.09	0.11	0.05	0.07	0.10	0.12	0.05	0.08	0.10	0.13
	TN	0.01	0.02	0.02	0.03	0.01	0.02	0.03	0.03	0.01	0.02	0.03	0.03
	SC	0.05	0.07	0.09	0.10	0.05	0.07	0.10	0.12	0.05	0.08	0.10	0.13
	NC	0.05	0.08	0.10	0.11	0.06	0.08	0.10	0.12	0.06	0.08	0.11	0.13
	MS	0.01	0.01	0.01	0.01	0.01	0.01	0.01	0.01	0.01	0.01	0.01	0.02
	GA	0.14	0.20	0.26	0.31	0.14	0.22	0.28	0.35	0.15	0.23	0.30	0.38
	AL	0.10	0.15	0.19	0.24	0.11	0.16	0.21	0.26	0.11	0.17	0.23	0.28
	FL	0.01	0.01	0.01	0.01	0.01	0.01	0.01	0.02	0.01	0.01	0.02	0.02
	Sum	0.42	0.61	0.77	0.92	0.44	0.64	0.84	1.03	0.45	0.68	0.90	1.12

Note: VA: Virginia, TN: Tennessee, SC: South Carolina, NC: North Carolina, MS: Mississippi, GA: Georgia, AL: Alabama, FL: Florida

Table S6. Statistic data of estimated log production of loblolly pine under different scenarios in the southeastern US.

Scenario	Total roundwood production each rotation (dry mass Million tonnes)
Low-density w/o thinning for S1–S3	485.2
Low-density w/ thinning for S1–S3	451.7
High-density w/ thinning for S1–S3	478.9
Annual aboveground harvest [a]	53.52

[a] was extracted from the U.S. Department of Agriculture Forest Service EVALIDator 2.0.3 online database (6). It sums up the annual aboveground harvest of loblolly pine from the study regions in the southern US including Alabama, Georgia, Mississippi, North Carolina, South Carolina, Florida, Tennessee, and Virginia states.

Table S7. Spatial datasets used in this study.

Data used	Name of dataset	Original resolution	Model components	Source
Site Index for loblolly pine	gSSURGO (July 2020)	30m	Forest growth model	(5)
Soil organic carbon content	SoilGrids (May 2020)	30m	RothC	(120)
Soil clay content	SoilGrids (May 2020)	30m	RothC	(120)
Climate data (monthly mean temperature, monthly precipitation, and monthly evaporation)	CRU TS v4.05 (1991–2020)	0.5 degree	RothC	(121)

Table S8. Inputs and assumptions for forest operations.

Parameter	Unit	Value
Rotation length	year	25
First fertilizer application time (93)	year	10
Second fertilizer application time (93)	year	16
Diesel consumption of site preparation and planting (77, 80)	kg ha^{-1}	95.5
Diesel consumption of applying fertilizers and herbicides (80)	kg ha^{-1} [a]	7.50
Diesel consumption in logging (12)	kg m^{-3}	1.66
Diesel consumption of felling (81)	kg m^{-3}	0.31
Nitrogen fertilizer usage (93)	kg N ha^{-1}	103
Phosphorus fertilizer usage (93)	kg P_2O_5 ha^{-1}	12.8
Herbicide usage (glyphosate) (80)	kg ha^{-1}	1.36
Aboveground live tree moisture content (60)	% *dry basis*	83.0
Aboveground live tree wet density (60, 95, 96)	kg m^{-3}	817
Aboveground live tree carbon content (60, 68)	% *dry basis*	50.0

[a] 1 ha = 10,000 m^2

Table S9. The life-cycle GHG emissions of materials and fuels used in this study (22, 32, 122).

Product	Quantity	GHG emissions (kg CO_2e)
Upstream		
Urea	1 kg	3.3
Triple superphosphates	1 kg	1.7
Glyphosate	1 kg	10.8
Wood chipping at forest road	1 oven dry kg wood fed in	0.013
Transportation	1 t km	0.17
Electricity	1 kWh	0.61
Nitrogen	1 kg	0.44
Reinforcing steel	1 kg	2.0
Hot rolled steel	1 kg	1.9
Concrete	1 m^3	285.9
Landfill of waste steel	1 t	5.2
Melamine Formaldehyde	1 kg	4.5
Diesel*	1 kg	0.33
Natural gas*	1 kg	0.58
Gasoline*	1 kg	0.54
Emission factors of fuel combustion		
Diesel	1 kg	3.2
Natural gas	1 kg	2.7
Gasoline	1 kg	3.2

*The total life cycle GHG emissions of fuels are the summation of these values for upstream production and GHG emission of fuel combustion shown at the end of this table.

Table S10. Ecoinvent 3.6 cut-off database processes used (32).

Product	Process
Urea	market for urea, as N | Cutoff, U_GLO
Triple superphosphates	triple_superphosphate_production_phosphate_fertiliser_as_P2O5_Cutoff, U_RoW
Glyphosate	market_for_glyphosate_glyphosate_Cutoff, U_GLO
Wood chipping at forest road	market for wood chipping, chipper, mobile, diesel, at forest road | wood chipping, chipper, mobile, diesel, at forest road | Cutoff, U_GLO
Transportation	transport, freight, lorry 16–32 metric ton, EURO6 | transport, freight, lorry 16–32 metric ton, EURO6 | Cutoff, U_RoW
Electricity	electricity, high voltage, production mix | electricity, high voltage | Cutoff, U_SERC
Nitrogen	market_for_nitrogen_liquid_nitrogen_liquid_Cutoff_U_RoW
Melamine Formaldehyde	market for melamine formaldehyde resin | melamine formaldehyde resin | Cutoff, U_RoW
Reinforcing steel	market for reinforcing steel | reinforcing steel | Cutoff, U_GLO
Hot rolled steel	steel production, low-alloyed, hot rolled | steel, low-alloyed, hot rolled | Cutoff, U_RoW
Concrete	market for concrete, normal | concrete, normal | Cutoff, U_RNA
Landfill of waste steel	treatment of scrap steel, inert material landfill | scrap steel | Cutoff, U_RoW

Table S11. Parameters for lumber production.

Parameter	Unit	Value
Bark mass fraction (60)	%	13.3
Electricity consumption of sawing (12, 16, 17, 23, 94)	kWh m^{-3} log input	24.4
Electricity consumption of kiln drying and kiln heat generation (12–14, 16, 17, 23)	kWh m^{-3} lumber input	26.9
Electricity consumption of planing (12, 13, 16, 17, 23)	kWh m^{-3} lumber input	18.2
Diesel consumption of hauling materials (12, 60, 95, 96)	kWh m^{-3} dried lumber	3.6
Gasoline consumption of hauling materials (12, 60, 95, 96)	kg m^{-3} dried lumber	0.23
Wet sawdust mass fraction of sawing byproducts (12)	%	17.9
Lumber mill waste recovery rate (12, 13, 16, 17, 97–107)	%	50.0
Lumber target moisture content (12, 13, 15, 34, 37, 100, 101, 106, 108)	% (*dry basis*)	12.0
Overall energy efficiency for energy generation and drying (12, 14, 16, 17)	%	17.9
Lumber drying shrinkage (12, 100, 101, 109)	%	9.1
Planing byproduct mass percentage (12, 13, 16, 17, 110)	%	17.8
Average transportation distance from field to lumber mills (16)	km	92
Average transportation distance of lumber distribution (12)	km	213
Average hauling distance to landfill site (111–114)	km	251

Table S12. Parameters for CLT production and end-of-life.

Parameter	Unit	Value
Lumber Moisture content (36)	%	12
Resin (MF) for finger-jointing and pressing (34, 37, 108, 117)	kg/m^3 lumber input	6.1
Diesel consumption for hauling materials	kg/m^3 lumber input	0.85
Planing shavings percentage (34, 108)	%	4.0
End cutting waste percentage (34, 108)	%	12.8
Finger-jointing waste percentage (34, 108)	%	0.93
Total electricity consumption of CLT production (34, 108, 117, 118)	kWh/m^3 final CLT produced	113.8
Transportation distance from lumber mill to CLT producer (34, 108)	km	266
Transportation distance from CLT producer to a construction site (12, 34)	km	213
Transportation distance from construction site to landfill site (111–114)	km	251
CLT life span (123)	years	60

Table S13. Setting, input parameters, and variables for the PMRC model.

	Values
Stand variables	
Physiographic Regions	Piedmont/Lower Coastal Plain/Upper Coastal Plain
Stand Productivity Measurement	Site Index
Site Index (ft)	50–105
Stand Density (trees/acre)	450 for low-density plantations/900 for high-density plantations
Planning Horizon (years)	0–25 for commercial plantations/0–35 for conservation plantations
Is the Basal Area of the stand known?	No
Thinning	
Should a thinning be included in this scenario?	Yes for commercial plantations/No for conservation plantations
Thinning Age (year)	10
Density Removed by Selective Thinning (trees/acre)	25% of the total trees is for low-density plantations/50% of the total trees is for high-density plantations
Silviculture	
Should a fertilization be included?	Yes for commercial plantations/No for conservation plantations
Pounds of elemental Nitrogen (lbs N/acre)	193
Is Phosphorus (P) included?	Yes
Year to be fertilized	10
Uncertainty range	
Forest growth uncertainty range	±15%

Unit conversion factors: 1 ft = 0.3048 m, 1 acre = 0.4047 hectare, 1 lbs = 0.4536 kg

Table S14. Parameters used to estimate dry mass whole living standings, snags, removed trees from thinning, and litterfall of loblolly pine plantations.

Parameter	Values (7)	Parameter	Values (8)
a_1	0.923902	d_1	0.7399
a_2	0.862958	d_2	–2.0287
a_3	0.078592	d_3	12.0950
a_4	–0.438251	d_4	327.234
a_5	–0.391617	d_5	0.62455
b_1	0.075259	e_1	0.00837
b_2	1.599282	e_2	0.19665
b_3	0.055050	e_3	2.5869
b_4	0.179042	e_4	18.5717
b_5	–0.369679	e_5	0.97146
c_1	0.010244	f_1	1.007
c_2	1.874136	f_4	–4.929
c_3	1.448326	g_1	–2.007
c_4	–0.177850		

Table S15. Parameters for modeling wood waste landfilling.

Parameter	Unit	Value
DOC_f (25, 29, 115, 116)		0.55
MCF (25, 29, 115, 116)		0.75
F (25, 29, 115, 116)		0.50
OX (25, 29, 115, 116)		0.05
K (12, 25, 29, 115, 116)		0.03
Volume rate of CH_4 to CO_2 in landfill gas emissions (31)		1.60
R (30)	%	75%
Power generation efficiency by wood incineration (30)	%	30

Table S16. Average structural material usage for 1 m^2 floor area (42, 43).

Material	Unit	Steel & concrete building	CLT building
Steel frame	kg	30.8	27.7
Steel deck	kg	14.3	
Concrete	kg	230	
Reinforcing bar	kg	8.9	
CLT	kg		70

Table S17. Values of key parameters for biochar production

Parameter	Unit	Value
Feedstock carbon content (68)	%	50.0
C/H ratio (68)		8.5
C/O ratio (68)		1.2
Feedstock ash content (68)	%	0.76
Feedstock moisture content (60)	% *dry basis*	83.0
Pyrolysis time (50)	minutes	60
Pyrolysis temperature (50)	°C	500
Pyrolysis pressure (50)	atm	1
Pyrolysis nitrogen flow (50)	% of inlet feedstock flow	16.7
Pyrolysis thermal efficiency (124)	%	90
Combustor excess air portion (125)	%	30
Diesel consumption in the wheel loader (119)	kg $ODMT^{-1}$ feedstock	1.4
Electricity consumption in the grinder (119)	kWh $ODMT^{-1}$ fed in	40
Electricity consumption in the hammer mill (119)	kWh $ODMT^{-1}$ fed in	33
Electricity consumption in the rotary drum dryer (119)	kWh $ODMT^{-1}$ fed in	45
Electricity consumption in the feed hopper (119)	kWh $ODMT^{-1}$ fed in	1.7
Transportation distance from the forest to the biochar plant (119)	km	69.5
Transportation distance from the biochar plant to the application field (32)	km	118

SI References

1. S. C. Cook-Patton, *et al.*, Lower cost and more feasible options to restore forest cover in the contiguous United States for climate mitigation. *One Earth* **3**, 739–752 (2020).
2. K. Coleman, D. S. Jenkinson, "RothC-26.3 - A Model for the turnover of carbon in soil" (2014).
3. IPCC, "Climate Change 2021: The Physical Science Basis AR 6 Work Group I" (2022).
4. W. M. Harrison, B. E. Borders, 1996 Yield prediction and growth projection for site-prepared loblolly pine plantations in the Carolinas, Georgia, Alabama and Florida. *PMRC Tech. Rep.*, 66 (1996).
5. Soil Survey Staff, Gridded Soil Survey Geographic (gSSURGO) Database for the Conterminous United States. United States Department of Agriculture, Natural Resources Conservation Service. *Available online at: https://gdg.sc.egov.usda.gov/.*
6. USDA Forest Service, U.S. Department of Agriculture Forest Service EVALIDator 2.0.3 online database, https://apps.fs.usda.gov/fiadb-api/evalidator (2022).
7. C. A. Gonzalez-Benecke, *et al.*, Local and general above-stump biomass functions for loblolly pine and slash pine trees. *For. Ecol. Manage.* **334**, 254–276 (2014).
8. C. A. Gonzalez-Benecke, E. J. Jokela, T. a Martin, Modeling the Effects of Stand Development, Site Quality, and Silviculture on Leaf Area Index, Litterfall, and Forest Floor Accumulations in Loblolly and Slash Pine Plantations. *For. Sci.* **58**, 457–471 (2012).
9. T. J. Albaugh, H. L. Allen, L. W. Kress, Root and stem partitioning of Pinus taeda. *Trees* **20**, 176–185 (2006).
10. M. L. Mobley, *et al.*, Surficial gains and subsoil losses of soil carbon and nitrogen during secondary forest development. *Glob. Chang. Biol.* **21**, 986–996 (2015).
11. J. C. Jenkins, D. C. Chojnacky, L. S. Heath, R. A. Birdsey, National-scale biomass estimators for United States tree species. *For. Sci.* **49**, 12–35 (2003).
12. K. Lan, S. S. Kelley, P. Nepal, Y. Yao, Dynamic life cycle carbon and energy analysis for cross-laminated timber in the Southeastern United States. *Environ. Res. Lett.* **15**, 124036 (2020).
13. R. D. Bergman, S. A. Bowe, Environmental impact of manufacturing softwood lumber in northeastern and north central United States. *Wood Fiber Sci.* **42**, 67–78 (2010).
14. R. A. Ananias, *et al.*, Energy Consumption in Industrial Drying of Radiata Pine. *Dry. Technol.* **30**, 774–779 (2012).
15. R. Bergman, "Chapter 13 - Drying and Control of Moisture Content and Dimensional Changes" in *Wood Handbook - Wood as an Engineering Material*, (2010), pp. 1–20.
16. M. R. Milota, C. D. West, I. D. Hartley, Gate-to-gate life-cycle inventory of softwood lumber production. *Wood Fiber Sci.* **37**, 47–57 (2005).
17. M. R. Milota, "CORRIM : Phase I Final Report Module B Softwood Lumber - Pacific Northwest Region" (2004).
18. J. L. Bowyer, R. Shmulsky, J. G. Haygreen, *Forest products and wood science: an introduction* (Blackweel Publishing, 2003).
19. US Environmental Protection Agency, Methodology for Thermal Efficiency and Energy Input Calculations and Analysis of Biomass Cogeneration Unit Characteristics. *Clean Air* **1**, 1–31 (2007).
20. S. Kumar, P. Ghosh, Sustainable bio-energy potential of perennial energy grass from reclaimed coalmine spoil (marginal sites) of India. *Renew. Energy* **123**, 475–485 (2018).
21. A. Demirbas, Calculation of higher heating values of biomass fuels. *Energy Sources, Part A Recover. Util. Environ. Eff.* **38**, 2693–2697 (2016).
22. Argonne National Laboratory, "The greenhouse gases, regulated emissions, and energy use in technologies (GREET) model" (2021).
23. M. Milota, CORRIM REPORT: Module C-Life Cycle assessment for the production of southeastern softwood lumber (2015).
24. S. David, *et al.*, "STUDY OF LIFE EXPECTANCY" (2007).
25. R. Pipatti, *et al.*, "Chapter 3: Solid Waste Disposal" in *2006 IPCC Guidelines for National Greenhouse Gas Inventories*, (2006), pp. 6.1-6.49.
26. C. Brandstätter, D. Laner, J. Fellner, C- and N- balances from a landfill aeration experiment in *The 6th International Workshop on Hydro- Physico-Mechanics of Landfills*, (2015), pp. 81–84.

27. S. A. Q. Burton, I. A. Watson-Craik, Ammonia and nitrogen fluxes in landfill sites: Applicability to sustainable landfilling. *Waste Manag. Res.* **16**, 41–53 (1998).
28. US EPA, Landfill Methane Outreach Program (LMOP) (2022).
29. IPCC, "Chapter 5 Waste" in *Good Practice Guidance and Uncertainty Management in National Greenhouse Gas Inventories*, (2000).
30. M. Anshassi, H. Sackles, T. G. Townsend, A review of LCA assumptions impacting whether landfilling or incineration results in less greenhouse gas emissions. *Resour. Conserv. Recycl.* **174**, 105810 (2021).
31. X. Chai, *et al.*, Characteristics of environmental factors and their effects on CH4 and CO2 emissions from a closed landfill: An ecological case study of Shanghai. *Waste Manag.* **30**, 446–451 (2010).
32. G. Wernet, *et al.*, The ecoinvent database version 3 (part I): overview and methodology. *Int. J. Life Cycle Assess.* **21**, 1218–1230 (2016).
33. L. Muszynski, "Effective Bonding Parameters for Hybrid Cross-Laminated Timber (CLT).," Oregon State University. (2017).
34. C. X. Chen, F. Pierobon, I. Ganguly, Life Cycle Assessment (LCA) of Cross-Laminated Timber (CLT) produced in Western Washington: The role of logistics and wood species mix. *Sustainability* **11** (2019).
35. Z. Zhang, K. Lan, Understanding the impacts of plant capacities and uncertainties on the techno-economic analysis of cross-laminated timber production in the southern u.s. *J. Renew. Mater.* **10**, 53–73 (2022).
36. ANSI/APA, "ANSI/APA PRG 320-2019 Standard for Performance-Rated Cross-Laminated Timber" (2019).
37. M. Gu, "Strength and Serviceability Performances of Southern Yellow Pine Cross-Laminated Timber (CLT) and CLT-Glulam Composite Beam," Clemson University. (2017).
38. R. Brandner, G. Flatscher, A. Ringhofer, G. Schickhofer, A. Thiel, Cross laminated timber (CLT): overview and development. *Eur. J. Wood Wood Prod.* **74**, 331–351 (2016).
39. L. Hasburgh, *et al.*, Effect of adhesives and ply configuration on the fire performance of Southern pine cross-laminated timber in *WCTE 2016 - World Conference on Timber Engineering*, (2016).
40. G. Wang, "Hygrothermal Performance of Southern Pine Cross-laminated Timber," North Carolian State University. (2018).
41. M. Popovski, I. Gavric, Performance of a 2-Story CLT House Subjected to Lateral Loads. *J. Struct. Eng. (United States)* **142**, 1–12 (2016).
42. B. D'Amico, F. Pomponi, J. Hart, Global potential for material substitution in building construction: The case of cross laminated timber. *J. Clean. Prod.* **279**, 123487 (2021).
43. M. C. Caruso, C. Menna, D. Asprone, A. Prota, LCA-Based Comparison of the Environmental Impact of Different Structural Systems. *IOP Conf. Ser. Mater. Sci. Eng.* **442** (2018).
44. K. Kenney, *et al.*, "Feedstock Supply System Design and Economics for Conversion of Lignocellulosic Biomass to Hydrocarbon Fuels: Conversion Pathway: Biological Conversion of Sugars to Hydrocarbons The 2017 Design Case (No. INL/EXT-13-30342)" (2013).
45. K. Lan, L. Ou, S. Park, S. S. Kelley, Y. Yao, Life Cycle Analysis of Decentralized Preprocessing Systems for Fast Pyrolysis Biorefineries with Blended Feedstocks in the Southeastern United States. *Energy Technol.*, 1900850 (2019).
46. M. Liao, S. Kelley, Y. Yao, Generating Energy and Greenhouse Gas Inventory Data of Activated Carbon Production Using Machine Learning and Kinetic Based Process Simulation. *ACS Sustain. Chem. Eng.* **8**, 1252–1261 (2020).
47. A. Anca-Couce, R. Scharler, Modelling heat of reaction in biomass pyrolysis with detailed reaction schemes. *Fuel* **206**, 572–579 (2017).
48. E. Ranzi, *et al.*, Chemical kinetics of biomass pyrolysis. *Energy and Fuels* **22**, 4292–4300 (2008).
49. P. E. A. Debiagi, *et al.*, Extractives Extend the Applicability of Multistep Kinetic Scheme of Biomass Pyrolysis. *Energy and Fuels* **29**, 6544–6555 (2015).
50. G. Liu, M. Li, B. Zhou, Y. Chen, S. Liao, General indicator for techno-economic assessment of renewable energy resources. *Energy Convers. Manag.* **156**, 416–426 (2018).
51. D. Woolf, *et al.*, Greenhouse Gas Inventory Model for Biochar Additions to Soil. *Environ. Sci. Technol.* **55**, 14795–14805 (2021).

52. A. R. Zimmerman, Abiotic and microbial oxidation of laboratory-produced black carbon (biochar). *Environ. Sci. Technol.* **44**, 1295–1301 (2010).
53. L. Leng, H. Huang, H. Li, J. Li, W. Zhou, Biochar stability assessment methods: A review. *Sci. Total Environ.* **647**, 210–222 (2019).
54. Y. Fang, B. Singh, B. P. Singh, E. Krull, Biochar carbon stability in four contrasting soils. *Eur. J. Soil Sci.* **65**, 60–71 (2014).
55. S. Chen, Y. Huang, J. Zou, Y. Shi, Mean residence time of global topsoil organic carbon depends on temperature, precipitation and soil nitrogen. *Glob. Planet. Change* **100**, 99–108 (2013).
56. Y. Liu, *et al.*, Residence time of carbon in paddy soils. *J. Clean. Prod.* **400**, 136707 (2023).
57. Y. Yan, X. Zhou, L. Jiang, Y. Luo, Effects of carbon turnover time on terrestrial ecosystem carbon storage. *Biogeosciences* **14**, 5441–5454 (2017).
58. Y. Yan, Y. Luo, X. Zhou, J. Chen, Sources of variation in simulated ecosystem carbon storage capacity from the 5th Climate Model Intercomparison Project (CMIP5). *Tellus, Ser. B Chem. Phys. Meteorol.* **66** (2014).
59. FAO, "Global Soil Organic Carbon Sequestration Potential Map – GSOCseq v.1.1 Technical mannual" (2022) https:/doi.org/10.4060/cb9002en.
60. P. D. Miles, W. B. Smith, Specific Gravity and Other Properties of Wood and Bark for 156 Tree Species Found in North America. *Res. Note. NRS-38*, 35 (2009).
61. M. Phanphanich, S. Mani, Impact of torrefaction on the grindability and fuel characteristics of forest biomass. *Bioresour. Technol.* **102**, 1246–1253 (2011).
62. M. D. Casal, M. V. Gil, C. Pevida, F. Rubiera, J. J. Pis, Influence of storage time on the quality and combustion behaviour of pine woodchips. *Energy* **35**, 3066–3071 (2010).
63. A. H. Zacher, *et al.*, Pyrolysis of woody residue feedstocks: Upgrading of bio-oils from Mountain-pine-beetle-killed trees and hog fuel. *Energy and Fuels* **28**, 7510–7516 (2014).
64. M. D. Westbrook Jr., W. Dale Greene, R. L. Izlar, Utilizing forest biomass by adding a small chipper to a tree-length southern pine harvesting operation. *South. J. Appl. For.* **31**, 165–169 (2007).
65. A. Mandalika, *et al.*, Potential of natural-origin loblolly pine tree fractions as a bioenergy feedstock. *Wood Fiber Sci.* **51** (2019).
66. M. T. Reza, M. H. Uddin, J. G. Lynam, S. K. Hoekman, C. J. Coronella, Hydrothermal carbonization of loblolly pine: reaction chemistry and water balance. *Biomass Convers. Biorefinery* **4**, 311–321 (2014).
67. J. Park, J. Meng, K. H. Lim, O. J. Rojas, S. Park, Transformation of lignocellulosic biomass during torrefaction. *J. Anal. Appl. Pyrolysis* **100**, 199–206 (2013).
68. C. W. Edmunds, *et al.*, Blended Feedstocks for Thermochemical Conversion : Biomass Characterization and Bio-Oil Production From Switchgrass-Pine Residues Blends. *Front. Energy Res.* **6**, 1–16 (2018).
69. M. Phanphanich, S. Mani, Drying characteristics of pine forest residues. *BioResources* **5**, 108–120 (2010).
70. Z. Chen, *et al.*, Characteristics and kinetic study on pyrolysis of five lignocellulosic biomass via thermogravimetric analysis. *Bioresour. Technol.* **192**, 441–450 (2015).
71. F. A. López, T. A. Centeno, I. García-Díaz, F. J. Alguacil, Textural and fuel characteristics of the chars produced by the pyrolysis of waste wood, and the properties of activated carbons prepared from them. *J. Anal. Appl. Pyrolysis* **104**, 551–558 (2013).
72. A. I. Ferreiro, *et al.*, Unresolved Issues on the Kinetic Modeling of Pyrolysis of Woody and Nonwoody Biomass Fuels. *Energy and Fuels* **31**, 4035–4044 (2017).
73. D. López-González, M. Fernandez-Lopez, J. L. Valverde, L. Sanchez-Silva, Thermogravimetric-mass spectrometric analysis on combustion of lignocellulosic biomass. *Bioresour. Technol.* **143**, 562–574 (2013).
74. V. B. Silva, A. Rouboa, Using a two-stage equilibrium model to simulate oxygen air enriched gasification of pine biomass residues. *Fuel Process. Technol.* **109**, 111–117 (2013).
75. G. Wang, T. Pinto, M. Costa, Investigation on ash deposit formation during the co-firing of coal with agricultural residues in a large-scale laboratory furnace. *Fuel* **117**, 269–277 (2014).

76. J. Daystar, C. Reeb, R. Venditti, R. Gonzalez, M. E. Puettmann, Life-Cycle Assessment of Bioethanol from Pine Residues via Indirect Biomass Gasification to Mixed Alcohols*. *For. Prod. J.* **62**, 314–325 (2012).
77. M. Puettmann, E. Oneil, M. Milota, L. Johnson, "Cradle to Gate Life Cycle Assessment of Softwood Lumber Production from the Southeast" (2013) https:/doi.org/10.13140/RG.2.2.12807.60327.
78. P. Dwivedi, J. R. R. R. Alavalapati, A. Susaeta, A. Stainback, Impact of carbon value on the profitability of slash pine plantations in the southern United States: an integrated life cycle and Faustmann analysis. *Can. J. For. Res.* **39**, 990–1000 (2009).
79. A. Kilpeläinen, A. Alam, H. Strandman, S. Kellomäki, Life cycle assessment tool for estimating net CO2 exchange of forest production. *GCB Bioenergy* **3**, 461–471 (2011).
80. D. Markewitz, Fossil fuel carbon emissions from silviculture : Impacts on net carbon sequestration in forests. *For. Ecol. Manage.* **236**, 153–161 (2006).
81. E. E. Oneil, *et al.*, Life-Cycle Impacts of Inland Northwest and Northeast / North Central Forest Resources. *Wood Fiber Sci.* **42**, 29–51 (2010).
82. D. Athanassiadis, G. Lidestav, I. Wästerlund, Fuel, Hydraulic Oil and Lubricant Consumption in Swedish Mechanized Harvesting Operations, 1996. *J. For. Eng.* **10**, 59–66 (1999).
83. P. Saud, J. Wang, W. Lin, B. D. Sharma, D. S. Hartley, A life cycle analysis of forest carbon balance and carbon emissions of timber harvesting in West Virginia. *Wood Fiber Sci.* **45**, 250–267 (2013).
84. A. Alam, A. Kilpeläinen, S. Kellomäki, Impacts of initial stand density and thinning regimes on energy wood production and management-related CO2 emissions in boreal ecosystems. *Eur. J. For. Res.* **131**, 655–667 (2012).
85. C. Whittaker, N. Mortimer, R. Murphy, R. Matthews, Energy and greenhouse gas balance of the use of forest residues for bioenergy production in the UK. *Biomass and Bioenergy* **35**, 4581–4594 (2011).
86. S. González-García, S. Berg, G. Feijoo, M. T. Moreira, Environmental impacts of forest production and supply of pulpwood: Spanish and Swedish case studies. *Int. J. Life Cycle Assess.* **14**, 340–353 (2009).
87. P. Mcnamee, *et al.*, An assessment of the torrefaction of North American pine and life cycle greenhouse gas emissions. *Energy Convers. Manag.* **113**, 177–188 (2016).
88. L. Johnson, B. Lippke, E. Oneil, Modeling Biomass Collection and Woods Processing Life-Cycle Analysis. *For. Prod. J.* **62**, 258–272 (2012).
89. T. Karjalainen, S. Kellomäki, A. Pussinen, Role of wood-based products in absorbing atmospheric carbon. *Silva Fenn.* **28** (1994).
90. T. Karjalainen, Greenhouse gas emissions from the use of primary energy in forest operations and long-distance transportation of timber in Finland. *Forestry* **69**, 215–228 (1996).
91. E. Lindholm, *Energy Use and Environmental Impact of Roundwood and Forest Fuel Production in Sweden* (2010).
92. R. Klvac, A. Skoupy, Characteristic fuel consumption and exhaust emissions in fully mechanized logging operations. *J. For. Res.* **14**, 328–334 (2009).
93. R. L. Amateis, J. Liu, M. J. Ducey, H. L. Allen, Modeling Response to Midrotation Nitrogen and Phosphorus Fertilization in Loblolly Pine Plantations. *South. J. Appl. For.* **24**, 207–212 (2000).
94. D. G. Devaru, R. Maddula, S. T. Grushecky, B. Gopalakrishnan, Motor-based energy consumption in west virginia sawmills. *For. Prod. J.* **64**, 33–40 (2014).
95. D. W. Patterson, P. F. Doruska, T. Posey, Weight and bulk density of loblolly pine plywood logs in southeast Arkansas. *For. Prod. J.* **54**, 145–149 (2004).
96. H. T. Patterson, A. I. Clark, Bulk density of southern pine logs. *For. Prod. J.* **38**, 36–40 (1988).
97. M. E. Plank, "Lumber recovery from ponderosa pine in the Black Hills, South Dakota /" (2014) https:/doi.org/10.5962/bhl.title.94229.
98. C. Liu, J. C. Ruel, A. Groot, S. Y. Zhang, Model development for lumber volume recovery of natural balsam fir trees in Quebec, Canada. *For. Chron.* **85**, 870–877 (2009).
99. C. E. Keegan III, T. A. Morgan, K. A. Blatner, J. M. Daniels, Trends in Lumber Processing in the Western United States. Part II: Overrun and Lumber Recovery Factors. *For. Prod. J.* **60**, 140–149 (2010).
100. R. D. Bergman, S. A. Bowe, Environmental impact of producing hardwood lumber using life-cycle inventory. *Wood Fiber Sci.* **40**, 448–458 (2008).

101. R. D. Bergman, S. A. Bowe, Life-cycle inventory of manufacturing hardwood lumber in southeastern US. *Wood Fiber Sci.* **44**, 71–84 (2012).
102. P. H. Steele, "Factors determining lumber recovery in sawmilling" (US Department of Agriculture, Forest Service, Forest Products Laboratory, 1984).
103. K. A. Kilborn, Lumber recovery studies of Alaska Sawmills, 1997 to 1999. *USDA For. Serv. - Gen. Tech. Rep. PNW*, 14–15 (2002).
104. H. Mickaël, A. Michaël, B. Fabrice, M. Pierre, D. Thibaud, Soil detritivore macro-invertebrate assemblages throughout a managed beech rotation. *Ann. For. Sci.* **64**, 219–228 (2007).
105. E. C. Lowell, D. W. Green, Lumber Recovery From Small-Diameter Ponderosa Pine From Flagstaff, Arizona in *USDA Forest Service Proceedings RMRS-P-22*, (2001), pp. 161–166.
106. S. Zhang, G. Chauret, Q. Tong, Impact of precommercial thinning on tree growth, lumber recovery and lumber quality in Abies balsamea. *Scand. J. For. Res.* **24**, 425–433 (2009).
107. F. Wagner, F. Taylor, Low lumber recovery at southern pine sawmills may be due to misshapen sawlogs. *For. Prod. J.* **43**, 53–55 (1993).
108. M. Puettmann, A. Sinha, I. Ganguly, "CORRIM Report - Life cyle assessment of cross laminated timber produced in Oregon" (2018).
109. E. Wang, T. Chen, S. Pang, A. Karalus, Variation in anisotropic shrinkage of plantation-grown pinus radiata wood. *Maderas Cienc. y Tecnol.* **10**, 243–250 (2008).
110. K. A. Blatner, C. E. Keegan, J. M. Daniels, T. A. Morgan, Trends in lumber processing in the western united states. part III: Residue recovered versus lumber produced. *For. Prod. J.* **62**, 429–433 (2012).
111. S. A. Thorneloe, K. A. Weitz, J. Jambeck, Moving from Solid Waste Disposal to Materials Management in the United States in *Proceedings of the Sardinia '05, International Solid and Hazardous Waste Symposium*, (2005).
112. S. A. Thorneloe, K. Weitz, J. Jambeck, Application of the US decision support tool for materials and waste management. *Waste Manag.* **27**, 1006–1020 (2007).
113. R. K. Ham, Overview and implications of u.s. sanitary landfill practice. *Air Waste* **43**, 187–190 (1993).
114. E. D. Wikramanayake, O. Ozkan, V. Bahadur, Landfill gas-powered atmospheric water harvesting for oilfield operations in the United States. *Energy* **138**, 647–658 (2017).
115. J. E. F. Jensen, R. Pipatti, "CH4 Emissions from Solid Waste Disposal" in *Good Practice Guidance and Uncertainty Management in National Greenhouse Gas Inventories*, (2006), pp. 339–348.
116. R. Pipatti, *et al.*, "Waste generation and compositon" in *2006 IPCC Guidelines for National Greenhouse Gas Inventories*, (2006).
117. Athena Sustainable Materials Institute;, A Life Cycle Assessment of Cross-Laminated Timber Produced in Canada. 37 (2013).
118. P. Bédard, *et al.*, Manufacturing Cross-Laminated Timber (CLT) Technological and Economic Analysis (2010).
119. K. Lan, *et al.*, Techno-Economic Analysis of Decentralized Preprocessing Systems for Fast Pyrolysis Biorefineries with Blended Feedstocks in the Southeastern United States. *Renew. Sustain. Energy Rev.*, 110881 (2021).
120. ISRIC, SoilGrids—global gridded soil information. *ISRIC-World Soil Inf.* (October 20, 2021).
121. H. Ian, T. J. Osborn, J. Phil, D. Lis, Version 4 of the CRU TS monthly high-resolution gridded multivariate climate dataset. 1–18 (2020).
122. US Environmental Protection Agency, "AP 42, Fifth Edition Compilation of Air Pollutant Emissions Factors" (2009).
123. H. Gu, R. Bergman, Life cycle assessment and environmental building declaration for the design building at the University of Massachusetts. *Gen. Tech. Rep. FPL-GTR-255. Madison, WI US Dep. Agric. For. Serv. For. Prod. Lab. 1-73.* **255**, 1–73 (2018).
124. V. Dornburg, A. P. C. Faaij, Efficiency and economy of wood-fired biomass energy systems in relation to scale regarding heat and power generation using combustion and gasification technologies. *Biomass and Bioenergy* **21**, 91–108 (2001).
125. N. Arena, J. Lee, R. Clift, Life Cycle Assessment of activated carbon production from coconut shells. *J. Clean. Prod.* **125**, 68–77 (2016).
